# Research on Move-to-Escape Enhanced Dung Beetle Optimization and Its Applications

**DOI:** 10.3390/biomimetics9090517

**Published:** 2024-08-29

**Authors:** Shuwan Feng, Jihong Wang, Ziming Li, Sai Wang, Ziyi Cheng, Hui Yu, Jiasheng Zhong

**Affiliations:** 1 School of Information, University of Michigan, Ann Arbor, MI 48105, USA; 2 Department of Mechanical Engineering, The University of Hong Kong, Hong Kong 999077, China; 3 Institute of Collaborative Innovation, University of Macau, Taipa 999078, Macau; 4 Tangshan Power Supply Company, State Grid Jibei Electric Power Company Limited, Tangshan 063000, China; 5 Department of Electrical and Electronic Engineering, The University of Manchester, Manchester M13 9PL, UK; 6 The School of Computer Engineering, Hubei University of Arts and Science, Xiangyang 441053, China; 7 Shenzhen Institutes of Advanced Technology, Chinese Academy of Sciences, Shenzhen 518000, China

**Keywords:** optimization algorithms, evolutionary algorithms, DBO, combinatorial optimization, discrete optimization

## Abstract

The dung beetle optimization (DBO) algorithm is acknowledged for its robust optimization capabilities and rapid convergence as an efficient swarm intelligence optimization technique. Nevertheless, DBO, similar to other swarm intelligence algorithms, often gets trapped in local optima during the later stages of optimization. To mitigate this challenge, we propose the Move-to-Escape dung beetle optimization (MEDBO) algorithm in this paper. MEDBO utilizes a good point set strategy for initializing the swarm’s initial population, ensuring a more uniform distribution and diminishing the risk of local optima entrapment. Moreover, it incorporates convergence factors and dynamically balances the number of offspring and foraging individuals, prioritizing global exploration initially and local exploration subsequently. This dynamic adjustment not only enhances the search speed but also prevents local optima stagnation. The algorithm’s performance was assessed using the CEC2017 benchmark suite, which confirmed MEDBO’s significant improvements. Additionally, we applied MEDBO to three engineering problems: pressure vessel design, three-bar truss design, and spring design. MEDBO exhibited an excellent performance in these applications, demonstrating its practicality and efficacy in real-world problem-solving contexts.

## 1. Introduction

In recent years, optimization problems have remained at the forefront, presenting significant challenges due to their complexity, often surpassing the scope of traditional mathematical approaches. Consequently, swarm intelligence (SI) algorithms have gained widespread popularity for their ease of implementation and simple frameworks. These algorithms draw inspiration from collective behaviors observed in nature, aiming to solve various optimization problems by simulating the cooperative, competitive, and adaptive behaviors of biological swarms. Metaheuristic algorithms have garnered widespread attention due to their simplicity, randomness, gradient-free nature, and ability to avoid local optima, which has motivated researchers to develop more competitive metaheuristic algorithms [1]. Currently, research on metaheuristic techniques mainly focuses on two themes: creating novel algorithms and improving existing well-known algorithms. There are three approaches to improving existing algorithms: combining two or more algorithms, modifying the search patterns of the algorithms, and introducing new operators into existing algorithms.

Based on their inspiration, metaheuristic algorithms can be categorized into two major classes: evolutionary algorithms and swarm-based methods. Evolutionary algorithms are a type of global optimization method inspired by the process of biological evolution in nature, known for their robustness and broad applicability. For instance, Particle Swarm Optimization (PSO) [2] is inspired by the concept of swarm cooperation, where particles traverse the search space by mimicking the foraging behavior of bird flocks to find optimal solutions. PSO has been successfully applied in areas such as neural network training and engineering design, demonstrating fast convergence and high-quality solutions. Harris Hawks Optimization (HHO) [3] emulates the cooperative hunting strategies of Harris’s hawks and has shown effectiveness in feature selection and energy management, with research highlighting its adaptability and robust performance. The Dragonfly Algorithm (DA) [4] mimics dragonflies’ behaviors like foraging and predator avoidance and has proven useful in tasks like image segmentation and job scheduling, effectively exploring complex search spaces. Similarly, the dung beetle optimization (DBO) [5] algorithm is inspired by the diverse behaviors of dung beetles and has been applied to environmental modeling and structural design, though it faces issues such as a slow initial search speed and limited exploration in later stages. Additionally, the recently developed Rao algorithm [6] is a remarkable algorithm, distinguished by its lack of metaphor and the absence of any algorithm-specific control parameters. Since its inception, the Rao algorithm has garnered significant attention and has been successfully applied to solve a variety of unconstrained and constrained problems [7].

These swarm intelligence (SI) algorithms have demonstrated remarkable efficiency and robustness in solving various optimization problems, offering novel approaches and methods for tackling complex problems. Their ability to simulate natural processes and collective behaviors allows them to adapt to diverse and dynamic problem environments, providing flexible and scalable solutions. The effectiveness of these algorithms has been validated across numerous domains, from engineering and computer science to economics and bioinformatics, showcasing their versatility and wide-ranging applicability.

Unfortunately, SI algorithms are not immune to shortcomings. Many of these algorithms suffer from several drawbacks, such as slower convergence speeds and diminished accuracy, particularly during the early stages of optimization. These limitations pose challenges to their widespread adoption across diverse domains. In response, scholars have undertaken targeted efforts to address these issues by refining SI algorithms, leading to the emergence of a variety of optimization algorithms [8,9]. These enhancements aim to bolster algorithmic performance significantly, overcoming the aforementioned hurdles and enhancing their utility in real-world applications. This iterative refinement process has been instrumental in advancing the effectiveness and applicability of SI algorithms.

In contemporary times, the persistent exploration of swarm intelligence (SI) algorithms has yielded substantial improvements in their efficacy, positioning them as indispensable tools for addressing practical challenges. Among the earliest SI algorithms, PSO and the Artificial Bee Colony (ABC) algorithm have emerged as prominent contenders, as well as the ant colony optimization (ACO) algorithm [10] that mimics the foraging of ant colonies. Researchers have leveraged these algorithms to tackle a diverse array of practical problems, spanning from devising hybrid multi-objective algorithms for optimizing pipeline scheduling [11] to employing PSO for software workload estimation [12] and utilizing PSO for controlling water-cooled units [13]. Multi-objective swarm intelligence optimization [14,15] not only significantly enhances algorithm performance but also improves efficiency and effectiveness in handling complex problems. Introducing new functions and improving algorithm parameters can also enhance algorithm performance, leading to more efficient and accurate solutions [16]. These applications underscore the adaptability and effectiveness of SI algorithms in real-world contexts, marking a significant advancement in their transition from theoretical constructs to indispensable problem-solving methodologies.

Heuristic algorithms have demonstrated robust problem-solving capabilities and adaptability across various fields. In context-aware dynamic service coordination for IoT environments, they optimize service selection and coordination, thereby enhancing system responsiveness and quality [17]. They also improve user classification and service recommendations in user clustering [18], significantly increasing text filtering accuracy in LSTM networks and thereby enhancing natural language processing performance [19]. In low-thrust reconfiguration for formation flying using Jordan normal form, these algorithms design more efficient trajectories, reducing fuel consumption [20]. For adaptive optimal control of affine nonlinear systems, they provide precise and stable control solutions through identifier–critic neural network approximation with relaxed persistent excitation conditions [21]. In network function virtualization, heuristic algorithms optimize resource allocation and reduce latency in service function chaining orchestration, thereby boosting network performance [22]. Additionally, in multi-objective robust optimization for MDVRPLS in refined oil distribution, they handle multiple objectives and uncertainties, offering more reliable and efficient distribution planning [23]. These cases demonstrate the broad applicability and effectiveness of heuristic algorithms in tackling complex optimization problems.

Metaheuristic algorithms have two important characteristics: exploitation and exploration [1]. Exploration refers to the global search capability, where strong exploration can help the algorithm thoroughly search unknown regions during the early iterations to locate a rough area of the global optimum. Exploitation is the local search capability, where strong exploitation aids the algorithm in finely searching already explored regions during later iterations to find promising solutions. Maintaining an appropriate balance between exploitation and exploration enables the algorithm to achieve outstanding performance, which is a challenge for swarm intelligence algorithms. All swarm intelligence algorithms attempt to balance these two attributes using various specialized operators and search patterns; however, research gaps still exist in this area. Applying algorithms to edge computing can be achieved by optimizing the allocation of computational resources, reducing latency, and improving energy efficiency [24,25,26].

To address the slow initial search speed and limited exploration encountered in the later stages of the DBO algorithm, this study presents an improved version named MEDBO. A key contribution of our work is the introduction of the novel walk function, inspired by the movements of rolling dung beetles and the evasive behaviors of foraging dung beetles. The walk function is designed to enhance the algorithm’s performance and efficacy by broadening the search scope and facilitating exploration within the search space. Moreover, this function is highly adaptable and not limited to DBO; it can be integrated into other metaheuristic algorithms to improve their overall performance as well. In addition to the lateral movement strategy, we develop an intelligent evasion mechanism that enables the algorithm to dynamically respond to competitive scenarios. These enhancements significantly boost the efficiency and accuracy of the global search process. Through these refinements, MEDBO demonstrates superior capabilities in overcoming the challenges faced by the original DBO algorithm, providing more reliable solutions for complex optimization problems in practical applications.

The innovations in this paper are as follows:

- We introduce a lateral movement strategy inspired by rolling dung beetles, aimed at expanding the search scope and promoting exploration within the search space, thereby preventing DBO from being trapped in local optima.
- We introduce the walk function to significantly enhance the search speed of DBO, thereby improving its ability to find optimal solutions, particularly in complex problem scenarios. The walk function is not only effective for the DBO algorithm but also adaptable for integration into other algorithms, demonstrating its broad applicability and potential to boost overall algorithm performance.
- Additionally, we incorporate the escape behavior of dragonflies, balancing the issues of exploitation and exploration in DBO, thereby better addressing competitive situations and enhancing the algorithm’s global search capabilities.

The structure of this paper is as follows: The first section introduces the development of metaheuristic algorithms and the advantages and disadvantages of the DBO algorithm. The second section details the steps of the DBO algorithm. The third section discusses improvements to the DBO algorithm and introduces the MEDBO algorithm. The fourth section evaluates the MEDBO algorithm and other classic algorithms using the CEC2017 test functions, including mean ranking and performance comparisons. The fifth section demonstrates the engineering applications of the MEDBO algorithm, specifically testing the tension (compression) design problem, the three-bar truss-design problem, and the pressure vessel problem. The sixth section concludes this paper, summarizing the benefits of the MEDBO algorithm and suggesting potential future enhancements for the DBO algorithm.

## 2. DBO

To better address the problem, we first define the objective function and feasible solutions:

(1) $$\begin{array}{c} \min\{f(x)|x\in X,X\subseteq S\} \end{array}$$

The objective function is f(x), where *x* is a feasible solution and *X* is the set of feasible solutions. *S* is the solution space. Here, *X* represents the population matrix of all populations, expressed as

(2) $$X={\left[\begin{array}{cccc} x_{1}^{1} & x_{2}^{1} & \cdots & x_{dim}^{1}\\ x_{1}^{2} & x_{2}^{2} & \cdots & x_{dim}^{2}\\ \vdots & \vdots & \ddots & \vdots \\ x_{1}^{N} & x_{2}^{N} & \cdots & x_{dim}^{N} \end{array}\right]}_{N\times \dim}$$

where *N* is the population size and dim is the dimensionality of the problem. In this paper, we tested the algorithm on CEC2017 with N=30 and dim=30 or dim=100 to ensure the reliability of the algorithm testing. Each solution in the initial population is a point at a random position in the solution space, representing a potential solution. Therefore, *x* can be expressed as

(3) $$\begin{array}{c} x_{j}^{n}=lb+\mathrm{rand}\times \left(ub-lb\right),n=1,2,\ldots ,N\;\mathrm{and}\;j=1,2,\ldots ,\dim \end{array}$$

For lb and ub, we test the CEC2017 where IEEE sets the search range from −100 to 100, so lb=−100 and ub=100. The function rand generates a random number between 0 and 1, ensuring the randomness in initialization.

### 2.1. Rollerball Dung Beetle

In the wilderness, dung beetles grapple with the challenge of steering their dung balls in a straight line, all while enduring the scorching sun. To simulate this intricate behavior, Equation (4) as described in the original paper was utilized to meticulously adjust the position of the dung beetle as it rolls its precious cargo:

(4) $$\begin{array}{cc} \; & x_{i}\left(t+1\right)=x_{i}\left(t\right)+a\cdot k\cdot x_{i}\left(t-1\right)+b\cdot \Delta \;x\\ \; & \Delta \;x=\left|x_{i}\left(t\right)-X^{worst}\right| \end{array}$$

In the present study’s framework, the variable *t* stands for the ongoing iteration count, while $x_{i}\left(t\right)$ denotes the position information of the *i*th dung beetle at the *t*th iteration. The parameter *a* governs whether the dung beetle veers off its initial trajectory. It is probabilistically assigned a value of either 1 or −1, where 1 signifies no deviation and −1 indicates deviation. The parameter *k*, confined within the interval (0, 0.2], represents the defect factor, initially set to 0.1 in the original study. The constant *b*, ranging from 0 to 1, was allocated a value of 0.3 in this investigation. *a* and *k* are constants, and their multiplication with $x_{i}\left(t\right)$ will update the position of $x_{i}\left(t\right)$. $X_{\mathrm{worst}}$ refers to the position of the worst agent in the population, while Δ *x* simulates the influence of solar illumination. A larger Δ *x* implies that the dung beetle is farther from the light source.

In their natural habitat, when faced with obstacles, dung beetles exhibit a behavior reminiscent of a dance to adjust their rolling trajectory. To simulate this scenario, the original study introduced a probability-based method to determine whether dung beetles encounter obstacles while rolling their dung balls. Upon encountering such obstacles, a tangent function is utilized to calculate a new rolling direction, mimicking the dance-like movements of dung beetles. This process is incorporated into the updated equation Equation (5), governing the position of the rolling dung beetle. This dance-like behavior reflects the flexibility and adaptability of dung beetles when confronted with obstacles, while also providing a biological rationale for modeling their behavior:

(5) $$x_{i}\left(t+1\right)=x_{i}\left(t\right)+\tan\left(\theta \right)\left|x_{t}\left(t\right)-x_{i}\left(t-1\right)\right|$$

**Figure 1:**
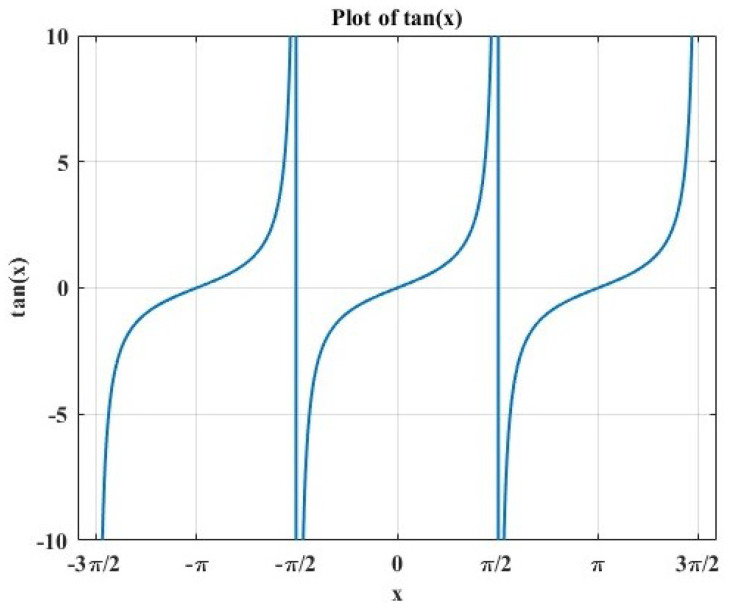
The value of tan(x).

And θ is the deflection angle belonging to [0,π]. The position is not updated when $\theta =0,\frac{\pi }{2}$ and π. tanθ approaching infinity causes $x_{i}\left(t\right)$ to become uncomputable. If θ approaches $\pi /2^{-}$, then tanθ approaches positive infinity; if θ approaches $\pi /2^{+}$, then tanθ approaches negative infinity. tanθ approaching infinity causes $x_{i}\left(t\right)$ to become uncomputable. If θ=π, tanθ equals 0, which results in $x_{i}\left(t+1\right)$ not being updated. The value of tan(x) is shown in Figure 1.

### 2.2. Spawning Dung Beetles

In their natural habitat, dung beetles meticulously select a secure location for oviposition. To mimic this behavior, the original paper introduces a boundary selection strategy to delineate this area, as shown in Equation (6):

(6) $$\begin{array}{cc} \; & Lb^{*}=\max\left(X^{best1}\times \left(1-R\right),Lb\right)\\ \; & Ub^{*}=\min\left(X^{best1}\times \left(1-R\right),Ub\right) \end{array}$$

Here, $Lb^{*}$ and $Ub^{*}$ represent the lower and upper limits of the spawning zone, respectively. $X_{\mathrm{best}1}$ refers to the position of the best agent in the population, while *R* is calculated as $R=1-t/T_{\max}$, with $T_{\max}$ representing the maximum number of iterations.

Upon discovering the optimal spawning region, dung beetles promptly deposit their eggs within it. Each spawning event entails a position adjustment, ensuring continuous exploration of the vicinity containing the current top solution and avoiding entrapment in local optima. The position adjustment for spawning dung beetles is dictated by Equation (7):

(7) $$x_{i}\left(t+1\right)=X^{best1}+b_{1}\times \left(x_{i}\left(t\right)-Lb^{*}\right)+b_{2}\times \left(x_{i}\left(t\right)-Ub^{*}\right)$$

In the document, $b_{1}$ and $b_{2}$ are stochastic variables with a scale of 1×Dim, where Dim serves as an indicator of the optimization problem’s complexity. $b_{1}$ is performing the Hadamard product with $\left(x_{i}\left(t\right)-Lb^{*}\right)$, and similarly, $b_{2}$ is performing the Hadamard product with $\left(x_{i}\left(t\right)-Ub^{*}\right)$.

### 2.3. Foraging Dung Beetles

Within their natural environment, dung beetles engaged in foraging also exhibit behaviors reminiscent of selecting secure locations, similar to when they lay eggs. This specific area is defined in the original text using the following Equation (8):

(8) $$\begin{array}{cc} \; & Lb^{b}=\max\left(X^{best2}\times \left(1-R\right),Lb\right)\\ \; & Ub^{b}=\min\left(X^{best2}\times \left(1-R\right),Ub\right) \end{array}$$

In this scenario, $X^{best2}$ symbolizes the optimal global position, while $Lb^{b}$ and $Ub^{b}$ establish the lower and upper boundaries of the optimal foraging zone, respectively. On the other hand, Lb and Ub represent the lower and upper limits for problem solving, respectively. Each foraging maneuver executed by the dung beetle triggers a singular position adjustment, guided by Equation (9):

(9) $$x_{i}\left(t+1\right)=x_{i}\left(t\right)+C_{1}\times \left(x_{i}\left(t\right)-Lb^{b}\right)+C_{2}\times \left(x_{i}\left(t\right)-Ub^{b}\right)$$

$C_{1}$ is a random variable following a normal distribution, while $C_{2}$ is a vector of size 1 × Dim, with its components uniformly distributed in the range [0, 1]. $C_{1}$ performs the Hadamard product with $\left(x_{i}\left(t\right)-Lb^{*}\right)$, and similarly, $C_{2}$ performs the Hadamard product with $\left(x_{i}\left(t\right)-Ub^{*}\right)$.

### 2.4. Stealing Dung Beetles

In nature, certain dung beetles choose to pilfer dung balls from their peers. To simulate this behavior, the original paper designates the optimal global location X as the position of the contested dung ball. The act of stealing, executed by the dung beetle engaged in this action, is depicted as a location update, formulated by the following equation:

(10) $$x_{i}\left(t+1\right)=X^{best2}+S\cdot g\cdot \left(\left|x_{i}\left(t\right)-X^{best1}\right|+\left|x_{i}\left(t\right)-X^{best2}\right|\right)$$

As outlined in the original paper, *S* is set as a constant with a fixed value of 0.5. The parameter *g* represents the magnitude of a random variable, while Dim is employed to signify the dimensionality of the problem being studied. *S* is a constant, *g* is a column vector, and $x_{i}\left(t\right)$ is also a vector. The dot product between them results in a vector. The manuscript specifies the population sizes for various categories of dung beetles: 6 for rollers, 6 for breeders, 7 for foragers, and 11 for thieves.

### 2.5. The Time Complexity of DBO

Firstly, in the initialization step, setting up the initial population and relevant parameters incurs a time complexity of O(N), where *N* represents the population size. Subsequently, within each iteration, the time complexity of updating the positions of all dung beetles, including rolling, spawning, foraging, and stealing dung beetles, remains O(N) per update. However, considering that the iterations in the algorithm constitute a loop, involving multiple updates across the entire population, the overall time complexity needs to account for the number of iterations $T_{\max}$. Therefore, the total time complexity is $O(T_{\max}\times N)$.

## 3. Enhanced DBO Algorithm with Added Walk Strategy

### 3.1. Our Motivation

As a novel algorithm, DBO has seen various improvements [27,28,29,30,31,32,33] up to May 2024. However, the algorithm is inherently constrained by the foraging behavior of dung beetles. Firstly, uncertainties in environmental conditions and resource distribution may lead to reduced search efficiency. Secondly, competition among individuals may result in some dung beetles being unable to obtain sufficient food, thereby affecting overall survival and reproductive success rates. Additionally, dung beetles exhibit relatively weak defense capabilities when confronted with predators or other dangers, rendering them susceptible to threats.

The original DBO algorithm suffers from drawbacks such as susceptibility to local optima, especially in handling complex optimization problems where convergence speed is slow. To overcome these limitations, we introduced the Move-to-Escape dung beetle optimization (MEDBO) algorithm. The key improvements of MEDBO lie in the introduction of a walking function and optimized foraging strategies. The walking function enables the algorithm to flexibly adjust step sizes and directions during the search, facilitating quicker escape from local optima. Meanwhile, optimized foraging strategies empower dung beetles to intelligently select suitable directions and strategies, accelerating the discovery of global optimal solutions. These enhancements not only expedite the convergence rate of the algorithm but also significantly enhance its efficiency and performance in tackling complex optimization problems. By introducing new strategies and adjusting parameters, MEDBO effectively addresses the limitations of foraging dung beetles, providing a more reliable and efficient approach to solving optimization problems.

The MEDBO algorithm addresses specific limitations in the original DBO algorithm, particularly in the context of dung beetle foraging behavior. In the original DBO, the foraging dung beetles exhibit incomplete food search behavior, which often leads to local optima and reduced search efficiency. To address this issue, we introduced a novel escape mechanism and a unique walk function. The escape mechanism helps dung beetles avoid local optima by dynamically responding to competitive scenarios, while the walk function significantly enhances the algorithm’s adaptability, enabling it to adjust step sizes and directions flexibly. Importantly, the walk function is original and has broad applicability, making it a valuable contribution that can be integrated into other algorithms as well. These innovative approaches, including the novel escape mechanism and the adaptable walk function, effectively address the limitations of the original DBO algorithm, improving its efficiency and performance in solving complex optimization problems.

### 3.2. Initial Parameter Definition

The process of dung beetles searching for food is dynamic. To derive their motion equations, we decompose projectile motion into two components: the horizontal (X-axis) and vertical (Y-axis). In the horizontal direction, dung beetles move uniformly in a straight line, and thus their motion Equation (11) is

(11) $$v=v_{0}+\int_{0}^{t}g\;dt=v_{0}+gt$$

In the vertical direction, the dung beetle undergoes uniform accelerated rectilinear motion, and its motion equation is as follows in Equation (12). We also assume ideal conditions with uniform acceleration. The distance between the dung beetle and the food is given by this formula, with the coordinate system set so that the initial distance $r_{0}=0$:

(12) $$\begin{array}{cc} \; & r=r_{0}+\int_{0}^{t}\left(v_{0}+gt\right)dt=r_{0}+v_{0}t+\frac{1}{2}gt^{2}\\ \; & r_{0}=0 \end{array}$$

Finally, the trajectory can be expressed as Equation (13):

(13) $$x_{i}\left(t+1\right)=\left[v_{0}\cos\left(\alpha \frac{t}{{\mathrm{Max}}_{i}\mathrm{ter}}\right)+\varepsilon \right]x_{\mathrm{best}\;}\left(t\right)+\left[v_{0}\sin\left(\alpha -\frac{\alpha t}{{\mathrm{Max}}_{i}\mathrm{ter}}\right)-g+\varepsilon \right]x_{i}\left(t\right)$$

**Figure 2:**
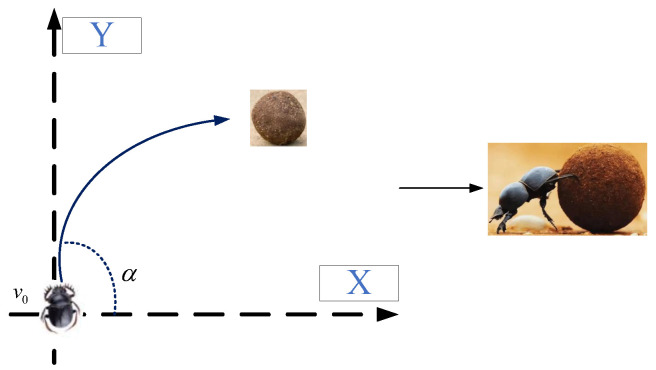
The components of the dung beetle’s foraging behavior.

Here, $x_{i}\left(t+1\right)$ represents the updated position of a search agent in the solution space at generation t+1, $x_{\mathrm{best}}\left(t\right)$ is the currently best-performing search agent, and $x_{i}\left(t\right)$ denotes the position of the current search agent. $Max_{iter}$ signifies the maximum number of iterations (generations), *t* denotes the current iteration, $v_{0}$ is fixed at 1 seg, α is set to $\frac{\pi }{2}$, ϵ is adjusted to 1 × 10^−6^, and g=9.807$\left(m/s^{2}\right)$. The foraging process is visualized in Figure 2. Equation (13) can be derived from Equations (14) and (15), as shown below:

(14) $$v_{0}=v_{0}\cos\left(\alpha \right)t\vec{j}+\left(\left(v_{0}\sin\left(\alpha \right)\right)t-\frac{1}{2}gt^{2}\right)\vec{k}$$

(15) $$v=\vec{r}=\left(v_{0}\cos\left(\alpha \right)\right)\vec{j}+\left(v_{0}\sin\left(\alpha \right)-gt\right)\vec{k}$$

### 3.3. Move-to-Escape Strategy

**Figure 3:**
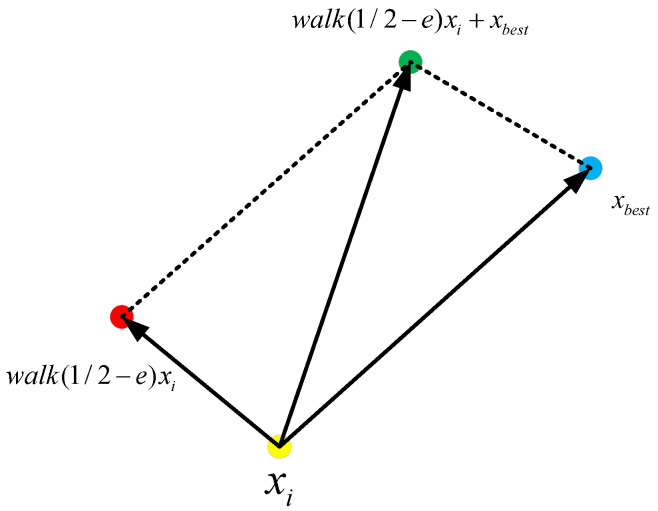
The optimal path combining the walk function.

In this strategy, dung beetles perform random rapid movements in the environment to search for food. To mathematically model this evasion strategy, we propose a function incorporating both local and global movements, described in Equation (16) and depicted in Figure 3. In this equation, walk$\left(\frac{1}{2}-e\right)\to x_{i}\left(t\right)$ represents local movement around $x_{i}\left(t\right)$, and adding $x_{best}\left(t\right)$ generates displacement in the solution search space (global movement):

(16) $$x_{i}\left(t+1\right)=x_{\mathrm{best}\;}\left(t\right)+\;\mathrm{walk}\;\left(\frac{1}{2}-e\right)x_{i}\left(t\right)$$

where $x_{i}\left(t+1\right)$ represents the new position of the search agent in the solution search space for generation t+1, $x_{\mathrm{best}}\left(t\right)$ is the best search agent for generation *t*, and “walk” is a random number generated between −1 and 1. *e* is a random number generated from a standard Cauchy distribution with mean 0 and standard deviation 1. $x_{i}\left(t\right)$ denotes the current *i*-th search agent in generation *t*. This behavior can be explained through Figure 3, where the dung beetle performs a central switch between the optimal position and the food, helping to avoid getting trapped in local optima.

### 3.4. Pseudocode for the MEDBO Algorithm

The pseudocode for the MEDBO algorithm is in Algorithm 1. The value of *a* is a uniformly distributed random real number greater than or equal to 0 and less than 1.
**Algorithm 1** Framework of the MEDBO Algorithm**Input:** Maximum iterations $T_{\max}$, population size *N*
**Output:** Optimal position $X^{best2}$ and its corresponding fitness value $f_{\min}$
 1:Initialize particle population (i=1,2,…,N) and relevant parameters. 2:**while** $t\leq T_{\max}$ **do** 3:   **for** *i* in rolling dung beetles **do** 4:   a=rand(1) 5:   **if** a≤0.9 **then** 6:      Update rolling dung beetle location with Equation (4). 7:   **else** 8:      Simulate rolling the ball with obstacles using Equation (5) for location update. 9:   **end if**10:   **end for**11:   Calculate nonlinear convergence factor $R=1-t/T_{\max}$.12:   **for** *i* in spawning dung beetles **do**13:   Update spawning dung beetle location using Equations (6) and (7).14:   **end for**15:   **for** *i* in foraging dung beetles **do**16:   Update velocity and angle using Equations (11) and (12).17:   Update foraging dung beetle location with Equation (13) and vectors *v*, *r*.18:   **end for**19:   **for** *i* in stealing dung beetles **do**20:   Construct walk function.21:   Update stealing dung beetle location using Equation (16).22:   **end for**23:**end while**24:**return** Optimal position $X^{best2}$ and corresponding fitness value $f_{\min}$.


### 3.5. The Time Complexity of MEDBO

Firstly, the initialization step involves setting up the initial particle population and relevant parameters, which typically incurs a time complexity of O(N). Each of these operations involves updating the location or parameters of the dung beetles and typically has a time complexity of O(N) per iteration. Since the algorithm iterates for a maximum of $T_{\max}$ iterations, the total time complexity for the iteration loop is $O(T_{\max}\times N)$. Finally, returning the optimal position and its corresponding fitness value has a constant time complexity of O(1). Therefore, the overall time complexity of the MEDBO algorithm is $O(T_{\max}\times N)$, reflecting its dependence on both the maximum number of iterations and the population size. We can determine that the time complexity of MEDBO is the same as that of DBO.

## 4. Experimental Results and Discussions

This study comprehensively evaluates the enhanced algorithm’s performance using the CEC2017 benchmark functions [34] as evaluation standards. The detailed specifications of these benchmark functions are delineated in Table 1. Within the CEC2017 dataset, the original F2 function was omitted, resulting in 29 single-objective benchmark functions for assessment. Among these, F1 and F2 exhibit single-peaked characteristics with a lone global minimum; F3–F9 present simple multi-modal functions featuring local minima; F10–F19 incorporate mixed functions comprising three or more CEC2017 benchmark functions post-rotation or -displacement; and F20–F29 encompass composite functions constructed from at least three mixed functions or CEC2017 benchmark functions post-rotation and -displacement.

The comparative algorithms enlisted are DBO, WOA, GWO, SAO, COA, and HHO. To ensure equitable experiments, the initial population size for all algorithms is standardized to 30, with a maximum of 500 iterations. To mitigate the impact of randomness, each algorithm undergoes 30 independent runs for statistical analysis. The algorithm parameters are shown in Table 2.

### 4.1. CEC2017 Test Function Results and Analysis

The statistical outcomes for the CEC2017 test functions in 30 and 100 dimensions were meticulously documented. These include the minimum (min), mean, and standard deviation (std) of each algorithm’s independent execution conducted 100 times. The best average result for each test function is highlighted in bold font. The last row, labeled “Total”, indicates the number of times each algorithm achieved the best result among all test functions. The statistical results for 30 and 100 dimensions are presented in Table 3 and Table 4, respectively.

From Table 3 and Table 4, it is evident from the comprehensive analysis that in the 30-dimensional scenario, MEDBO secured the optimal solution in 14 out of 29 test functions, showcasing a notably commendable performance. However, for the remaining 15 test functions, while the optimal solution was not reached, MEDBO still delivered satisfactory suboptimal results, underscoring its effectiveness in tackling diverse problems. Similarly, in the case of 100 dimensions, MEDBO achieved the best solution in 14 out of 29 test functions, and for the remaining 15 test functions, though optimal outcomes were not realized, relatively robust suboptimal results were obtained. This underscores the resilience and dependability of MEDBO in high-dimensional spaces. Below, detailed examination results are provided:

- On the unimodal function F1, MEDBO fell short of WOA in both the 30-dimensional and 100-dimensional cases, failing to achieve the best performance among all algorithms. However, on the unimodal function F2, MEDBO ranked fifth in both 30 and 100 dimensions, closely trailing SAO and WOA but significantly outperforming DBO. This indicates that the improvement effect of the MEDBO algorithm is remarkable.
- In the realm of simple multimodal problems F3–F9, MEDBO stands out in the 30-dimensional scenario, securing the top position in F3, F4, F6, F7, and F9, showcasing its remarkable performance. This indicates MEDBO’s capability to efficiently navigate through various multimodal functions and locate global optima effectively. However, it slightly falls behind WOA and SAO in F5 and F8. Nevertheless, it demonstrates a significant improvement overall. Transitioning to the 100-dimensional scenario, MEDBO maintains its dominance, leading the pack in F4, F5, F8, and F9, showcasing its superiority in high-dimensional spaces. While it trails WOA and SAO in some functions, its performance remains consistently impressive across most test functions, affirming the reliability and robustness of the MEDBO algorithm. Whether in 30 or 100 dimensions, it outperforms DBO, highlighting its substantial enhancement. The commendable effectiveness of the MEDBO algorithm in tackling multimodal optimization problems offers compelling support for addressing real-world challenges.
- For the hybrid functions F10–F19, in the 30-dimensional scenario, MEDBO ranked highest in F11, F15, and F17, demonstrating its excellent performance in finding optimal solutions. While it slightly trailed behind WOA in other functions, compared to the basic DBO, MEDBO still exhibited a superior performance. In the 100-dimensional scenario, although it slightly lagged behind WOA and SAO in some functions, MEDBO performed remarkably well in F11, F15, F16, and F19, showcasing its improved effectiveness. Thus, MEDBO’s performance in multi-dimensional spaces surpasses that of DBO, further emphasizing the significance of its improvement.
- In the case of composite functions F20–F29, with a dimension of 30, MEDBO ranks first in F20, F22–F24, and F26, indicating a relatively strong performance. However, its performance falls slightly short of WOA when compared. Nonetheless, compared to the basic DBO, MEDBO demonstrates a superior performance, showcasing higher efficiency and accuracy in solving composite function optimization problems. With the dimension increasing to 100, MEDBO continues to excel, ranking first in F20–F23, F26, and F28, underscoring its robustness in high-dimensional spaces.

### 4.2. CEC2017 Convergence Curve Analysis

**Figure 4:**
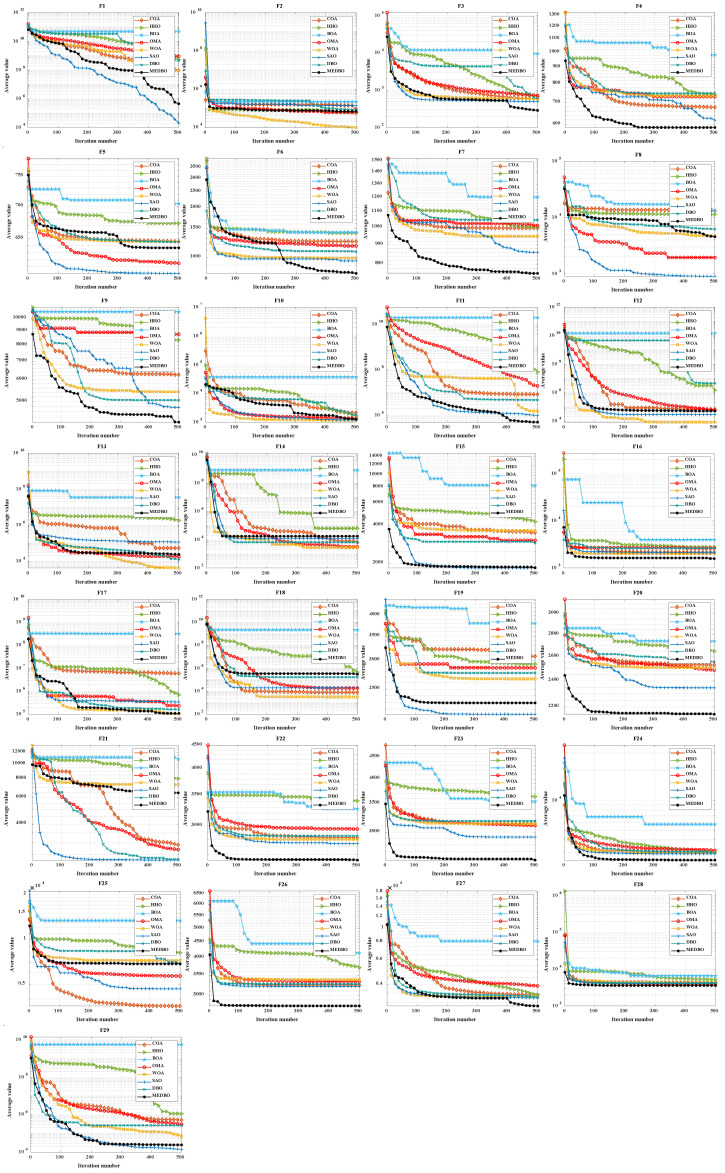
CEC2017 test curves chart (Dim = 30).

**Figure 5:**
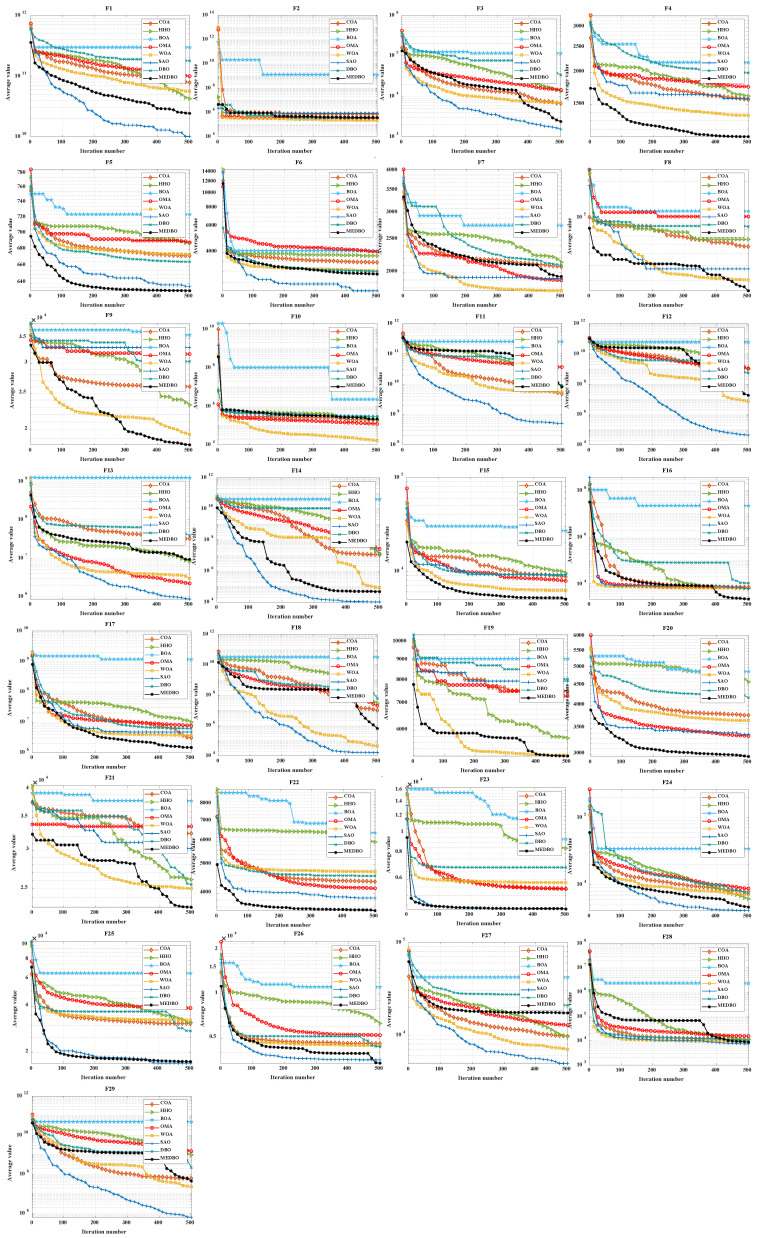
CEC2017 test curves chart (Dim = 100).

To evaluate both the accuracy and convergence speed of the algorithms, convergence curves were plotted for both 30 and 100 dimensions, depicting the performance of MEDBO alongside other algorithms, as shown in Figure 4 and Figure 5. Notably, each subplot represents the number of iterations on the horizontal axis and the average convergence curve of function test values based on 30 and 100 runs on the vertical axis. An analysis of the figures reveals the following observations:

- For the unimodal problem F1, MEDBO initially exhibited a slower convergence rate compared to SAO. However, approximately two-thirds into the iterations, MEDBO’s convergence rate accelerated, gradually catching up with SAO and ultimately surpassing other comparative algorithms. This trend was consistent across both 30-dimensional and 100-dimensional scenarios. In the case of the unimodal problem F2, MEDBO demonstrated notably faster convergence compared to DBO, resulting in superior outcomes for F2.
- For the simple multimodal functions F3–F9, MEDBO demonstrates an outstanding performance in 30 dimensions. At this dimensionality, MEDBO exhibits the fastest convergence speed and achieves the optimal solution for F3, F4, F6, F7, and F9. Particularly noteworthy is its performance on F6, where MEDBO reaches the equilibrium point of the DBO algorithm in just about 280 iterations, highlighting the remarkable effectiveness of MEDBO’s improvements. In 100 dimensions, MEDBO continues to excel, converging fastest on F4, F5, F8, and F9, and attaining optimal solutions as well. These results underscore the robustness and superiority of MEDBO in high dimensions.
- For the hybrid functions F10–F19, MEDBO has showcased an impressive convergence speed, with notable excellence observed in F15 and F19, where it consistently maintains a leading position. In 30 dimensions, MEDBO achieves the optimal solution fastest in F11, F15, F16, and F17. Notably, for F15 and F16, MEDBO reaches the equilibrium point before 100 iterations, surpassing other algorithms in solution accuracy. In 100 dimensions, MEDBO attains optimal solutions in F15, F16, F17, and F19, showcasing rapid solving speeds far surpassing those of the DBO algorithm.
- In the case of composite functions F20–F29, MEDBO consistently demonstrates a remarkable performance in 30 dimensions. It converges at an astounding pace, reaching optimal solutions in F20, F22, F23, F26–F29, far surpassing other algorithms. Only in F28, F29, and SAO does MEDBO perform similarly to others. This underscores MEDBO’s exceptional capability in tackling high-dimensional problems. In 100 dimensions, MEDBO continues to excel, achieving optimal solutions in F20–F23, F25, and F26, with impressive solving speeds. This further confirms MEDBO’s suitability and superiority in high-dimensional problem solving.

### 4.3. Wilcoxon Rank-Sum Test

The Wilcoxon rank-sum test, a robust nonparametric statistical method [40,41], is utilized for comparing the performance discrepancies between enhanced and traditional algorithms, avoiding assumptions about specific data distributions. In this study, we employed this method to analyze the variances between six different benchmark algorithms and MEDBO. By conducting experiments on the CEC2017 dataset across various dimensions, we obtained insightful results that shed light on the performance of different algorithms in tackling optimization problems.

In the 30-dimensional data (Table 5), MEDBO demonstrated significant performance advantages over WOA and SAO, likely due to its enhanced ability to explore the search space efficiently. Comparisons with DBO and HHO revealed significant differences across all test functions, highlighting its robustness and stability. However, comparisons with GWO and COA indicated MEDBO’s superiority in specific test functions, albeit with some performance fluctuations.

Further analysis of the results in the 100-dimensional data (Table 6) showed significant performance advantages of MEDBO over DBO, WOA, and SAO, suggesting its adaptability to high-dimensional problems. However, comparisons with GWO, COA, and HHO revealed significant differences in some functions, which may be attributed to different algorithms’ search strategies and convergence properties in high-dimensional spaces.

### 4.4. Comparison of MEDBO with Other Improved DBO Algorithms

We selected the improved DBO algorithms from 2023 up to June 2024 for comparison with our MEDBO algorithm. The test functions chosen include several samples from CEC2017: simple multimodal functions (F7), hybrid functions (F12 and F19), and composition functions (F26). The dimensions selected were 30 and 100, with each test independently repeated 100 times. We recorded the minimum value, mean, and variance and ranked the results based on the mean values to derive the final ranking results.

The statistical results are shown in Table 7 and Table 8. As seen in the 30-dimensional tests, the MEDBO algorithm consistently ranked first across the four different test functions, with relatively low variance, indicating a stable performance. In the 100-dimensional tests, MEDBO outperformed the other algorithms in three out of the four test functions. The only exception was in the hybrid functions (F12), where MEDBO slightly underperformed compared to the SCDBO algorithm but demonstrated better stability. Overall, the MEDBO algorithm maintained an optimal performance across the majority of test cases.

### 4.5. Analysis of Convergence Behavior

**Figure 6:**
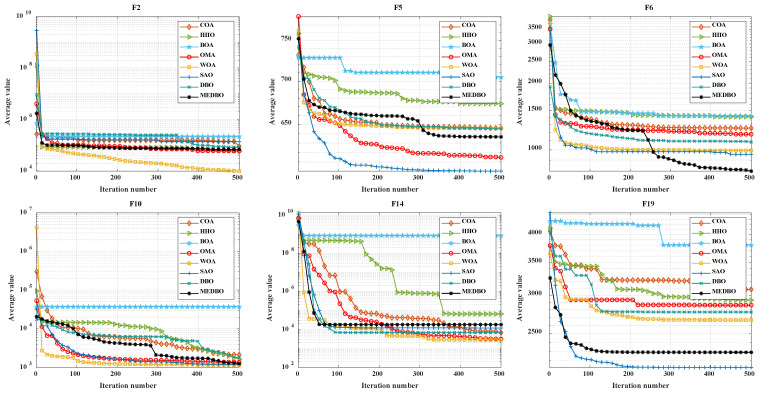

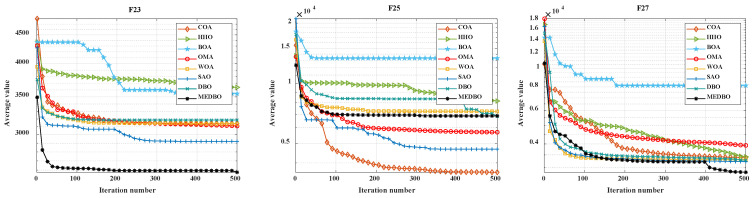
The convergence curves by the DBO algorithm and other optimizers on benchmark test functions (Dim = 30).

**Figure 7:**
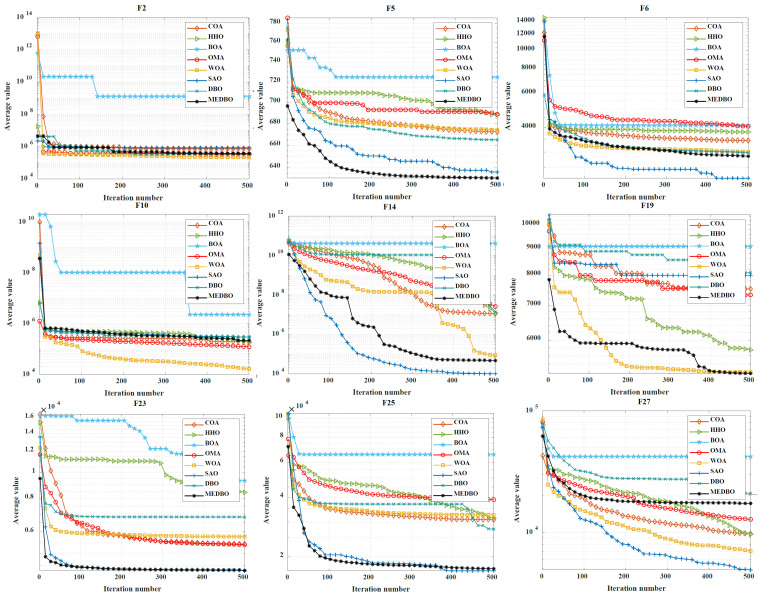
The convergence curves by the DBO algorithm and other optimizers on benchmark test functions (Dim = 100).

We conducted a detailed analysis of the convergence curves for the MEDBO algorithm and seven other optimization methods, as shown in Figure 6 and Figure 7. Figure 6 presents the convergence curves for a dimensionality of 30 (dim = 30), while Figure 7 presents the curves for a dimensionality of 100 (dim = 100). The horizontal axis represents the number of iterations, and the vertical axis represents the average best value obtained so far. To comprehensively evaluate the convergence performance of the MEDBO algorithm, we selected nine benchmark test functions, including one unimodal function (F2), two simple multimodal functions (F5 and F6), three hybrid functions (F10, F14, and F19), and three composition functions (F23, F25, and F27).

For F2, it can be observed that regardless of whether dim = 30 or dim = 100, the convergence performance of MEDBO is excellent, with the curve stabilizing at around 50 iterations, indicating a strong global search capability when handling unimodal functions. For the simple multimodal functions (F5 and F6), when the dimensionality is 30, the algorithm stabilizes after 400 iterations, while at dim = 100, the convergence speed is faster, reaching stability within 300 iterations. This suggests that in higher-dimensional simple functions, the convergence performance of MEDBO improves.

Regarding the hybrid functions (F14 and F19), at dim = 30, MEDBO converges particularly quickly, significantly outperforming other algorithms; however, at dim = 100, although the convergence speed is slightly reduced, it still effectively prevents getting trapped in local optima, demonstrating the effectiveness of our improvements. In the tests on composition functions (F23, F25, and F27), the average convergence curves of MEDBO stabilize at an early stage, further proving the superior convergence performance of the MEDBO algorithm.

These results indicate that, compared to state-of-the-art optimization techniques, the proposed MEDBO algorithm not only exhibits strong global search capabilities across various optimization problems but also shows good convergence characteristics in high-dimensional scenarios, achieving a higher success rate. These attributes make the MEDBO algorithm exhibit exceptional application potential in the field of optimization.

## 5. Engineering Optimization Issues

### 5.1. Tension (Compression) Design Problem

The design problem of tension/compression springs [42] is a crucial topic in the engineering field because springs play a key role in many mechanical systems. At the core of this design problem is the optimization of key parameters of the spring to ensure an optimal performance under various operating conditions. Optimizing parameters such as wire diameter, average coil diameter, and number of active coils aims to minimize the weight of the spring while ensuring appropriate tension or compression under different loading conditions. This is vital for numerous industries including automotive manufacturing, aerospace, and mechanical engineering, where springs are essential for various motion and mechanical functions.

When addressing this design problem, engineers must consider multiple factors including the material properties of the spring, environmental requirements, and system performance indicators. They must weigh different design options to find the optimal solution that meets the functional requirements while minimizing the weight of the system as much as possible to enhance efficiency and performance. Therefore, the design problem of tension/compression springs is not only an engineering challenge but also a test of engineers’ creativity and technical capabilities.

By solving this design problem, the engineering community can obtain lighter and more efficient spring-design solutions, thereby driving innovation and development in the engineering field. This not only contributes to improving product quality and performance but also helps to reduce production costs, promoting sustainable industry development. Hence, the design problem of tension/compression springs holds significant research and practical value in the engineering field, playing a pivotal role in advancing technological progress and engineering practices.

**Figure 8:**
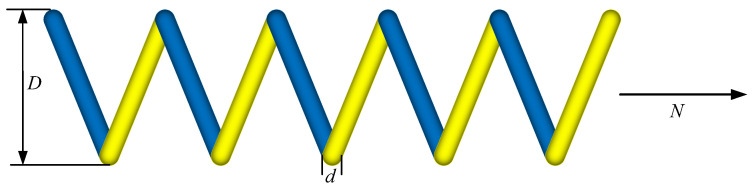
Extension (compression) spring-design issues.

Figure 8 illustrates the schematic diagram of the tension/compression spring-design problem, aiming to minimize the weight of the spring by adjusting parameters such as the wire diameter (*d*), average coil diameter (*D*), and number of active coils (*N*). The objective is to ensure that the spring achieves the desired performance under various operating conditions while minimizing its weight as much as possible. Such an optimization design not only enhances the efficiency and performance of the spring system but also reduces costs, providing more efficient and lightweight spring solutions for various applications.

Consider:

(17) $$x=\left[x_{1},x_{2},x_{3}\right]=\left[d,D,N\right],$$

Minimize:

(18) $$f\left(\vec{x}\right)=\left(x_{3}+2\right)x_{2}x_{1}^{2},$$

Subject to:

(19) $$\begin{array}{c} \begin{array}{c} g_{1}\left(\vec{x}\right)=1-\frac{x_{2}^{3}x_{3}}{71,755x_{1}^{4}}\leq 0\\ g_{2}\left(\vec{x}\right)=\frac{4x_{2}^{2}-x_{1}x_{2}}{12,566\left(x_{2}x_{1}^{3}-x_{1}^{4}\right)}+\frac{1}{5108x_{1}^{2}}\leq 0\\ g_{3}\left(\vec{x}\right)=1-\frac{140.45x_{1}}{x_{2}^{2}x_{3}}\leq 0\\ g_{4}\left(\vec{x}\right)=\frac{x_{1}+x_{2}}{1.5}-1\leq 0 \end{array} \end{array}$$

where $0.05\leq x_{1}\leq 2,0.25\leq x_{2}\leq 1.3,$ and $2\leq x_{3}\leq 15$.

**Figure 9:**
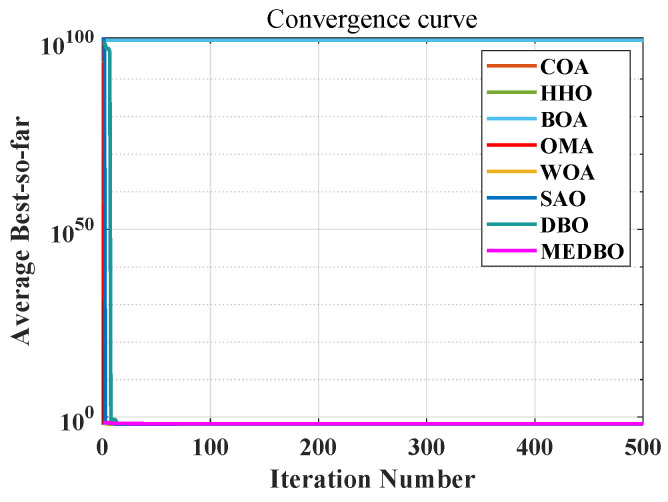
The tension (compression) spring-design convergence plot.

Figure 9 vividly illustrates the iterative process of MEDBO and other algorithms tackling the tension (compression) spring-design problem. Remarkably, MEDBO achieves the minimum value with the fewest iterations, showcasing its remarkable efficiency and superior performance in optimization endeavors. This efficiency is underpinned by MEDBO’s adaptive step-size adjustment, intelligent initialization strategy, efficient search approach, and dynamic updating mechanism. Such attributes empower MEDBO to swiftly converge, promptly pinpointing the optimal solution while drastically reducing computational resources and design time. In real-world engineering scenarios, this efficiency edge is pivotal for accelerating the pace and elevating the quality of design-optimization efforts, firmly establishing MEDBO as the premier choice for addressing intricate design challenges.

Table 9 presents the results of 100 repetitions of the tension (compression) spring-design problem experiment, highlighting the performance of different optimization algorithms. Among them, it is noticeable that the MEDBO algorithm exhibits the lowest cost, indicating its efficiency in finding optimal solutions for the design problem. This observation underscores the robustness and reliability of the MEDBO algorithm in tackling the complexities of the spring-design-optimization task. Such consistent superiority over multiple iterations reaffirms the algorithm’s potential for practical implementation in real-world engineering scenarios, where minimizing costs and maximizing performance are paramount concerns.

### 5.2. Three-Bar Truss-Design Problem

Confronting the task of designing a three-pole truss [43,44,45,46,47,48], engineers are tasked with weighing numerous considerations, spanning structural integrity, material expenses, and ecological impact. This undertaking transcends mere architectural design, evolving into a multifaceted engineering venture that melds mechanics, materials science, and mathematical modeling.

**Figure 10:**
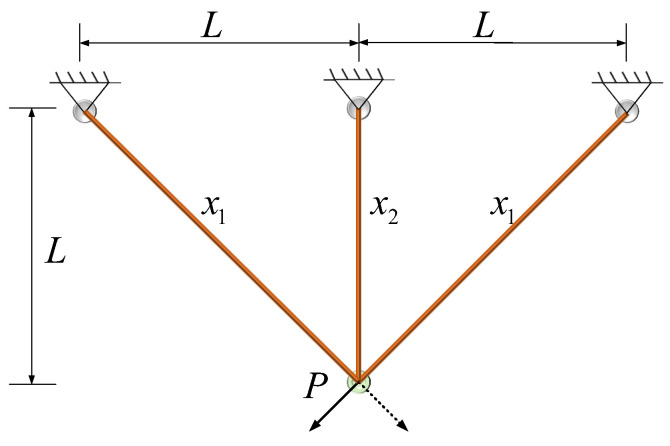
Triangle truss design.

Ensuring the stability and safety of the truss necessitates meticulous calculations of stress and deformation for each constituent, guaranteeing adherence to design specifications across diverse loading scenarios. Simultaneously, engineers must devise strategies to diminish the truss’s overall volume, thereby curbing material outlays and mitigating resource depletion. The truss with three poles in the design is illustrated in Figure 10.

This quest for engineering optimization assumes paramount significance in contemporary society. With resources dwindling and environmental concerns escalating, engineers are compelled to pioneer innovative solutions for crafting structures that are not only more efficient but also sustainable. Thus, by tackling the intricacies of three-pole truss design, they not only enhance engineering prowess but also champion the cause of future sustainability endeavors.

Consider variable:

(20) $$\begin{array}{c} x=(x_{1},x_{2}). \end{array}$$

Minimize:

(21) $$\begin{array}{c} \min f\left(x\right)=\left(2\sqrt{2}x_{1}+x_{2}\right)\times L. \end{array}$$

Subject to:

(22) $$\begin{array}{cc} \; & g_{1}=\frac{\sqrt{2}x_{1}+x_{2}}{\sqrt{2}x_{1}^{2}+2x_{1}x_{2}}P-\sigma \leq 0\\ \; & g_{2}=\frac{x_{2}}{\sqrt{2}x_{1}^{2}+2x_{1}x_{2}}P-\sigma \leq 0\\ \; & g_{3}=\frac{1}{x_{1}+\sqrt{2}x_{2}}P-\sigma \leq 0 \end{array}$$

where P=2,L=100,σ=2.

With bounds $0\leq x_{1},x_{2}\leq 2$.

**Figure 11:**
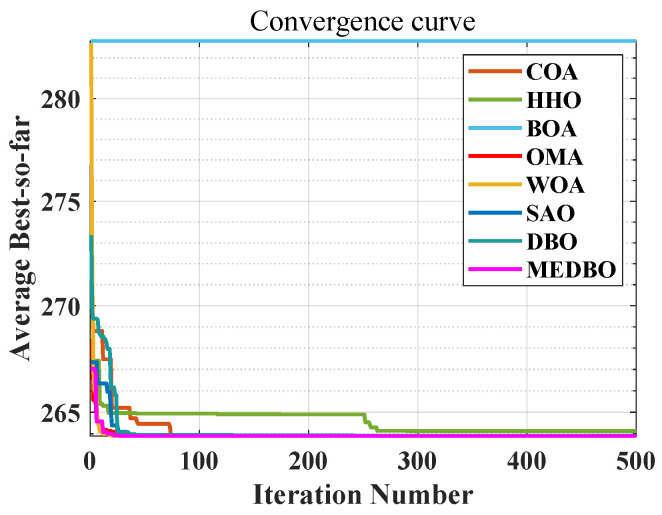
Three-bar truss convergence curve diagram.

The experimental results are compiled in Table 10, illustrating the performance of eight algorithms on the three-rod truss-design problem. Upon comparing the data in Table 10, it is evident that the MEDBO algorithm consistently ranks first in average performance and exhibits the lowest variance among the algorithms considered. Furthermore, observations from Figure 11 indicate that the MEDBO algorithm demonstrates the fastest iteration speed in solving the three-rod truss-design problem, achieving optimal solutions at the quickest rate.

These findings hold significant implications for the field of engineering design and optimization. Firstly, they underscore the notable advantages of the emerging MEDBO algorithm in addressing complex structural-design challenges. Its efficiency and accuracy provide engineers and researchers with more reliable design and optimization solutions. Additionally, the algorithm not only satisfies structural stress constraints but also concurrently reduces structural volume and costs, thereby offering more economical and feasible solutions for engineering projects.

Moreover, these discoveries offer valuable insights for future engineering practices. With ongoing advancements in engineering technology and increasing demands for efficient and precise design tools to meet evolving structural design requirements, the successful application of the MEDBO algorithm provides robust support for innovation and development in the engineering domain, paving the way for new possibilities in future engineering endeavors.

### 5.3. Pressure Vessel Problem

**Figure 12:**
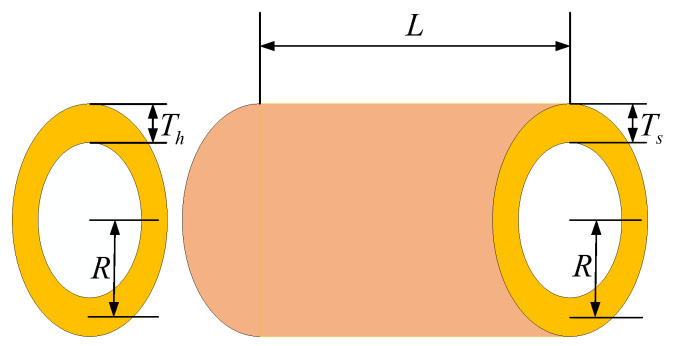
Pressure vessel parameter diagram.

The primary objective in pressure vessel design [49,50] is cost reduction while ensuring an optimal performance. This involves optimizing four critical variables: shell thickness ($T_{s}$), head thickness ($T_{h}$), inner radius (*R*), and the length of the cylindrical section excluding the heads (*L*), as depicted in Figure 12. Engineers must delicately balance these factors to preserve structural integrity and durability while minimizing expenses. Shell and head thickness directly impact vessel strength and pressure resistance, while inner radius and cylindrical section length affect internal volume and surface area, thus influencing manufacturing costs. Consequently, designers must achieve an optimal equilibrium among these parameters to meet performance requirements and minimize expenses. This entails a comprehensive consideration of material properties, manufacturing processes, safety standards, and cost factors to develop the most efficient design solution.

Consider variable:

(23) $$\begin{array}{c} \vec{x}=\left[x_{1}\;x_{2}\;x_{3}\;x_{4}\right]=\left[T_{s}\;T_{h}\;R\;L\right] \end{array}$$

Minimize:

(24) $$\begin{array}{c} f\left(\vec{x}\right)=0.6224x_{1}x_{3}x_{4}+1.7781x_{2}x_{3}^{2}+3.1661x_{1}^{2}x_{4}+19.84x_{1}^{2}x_{3} \end{array}$$

Subject to:

(25) $$\begin{array}{c} \begin{array}{c} g_{1}\left(\vec{x}\right)=-x_{1}+0.0193x_{3}0\\ g_{2}\left(\vec{x}\right)=-x_{3}+0.00954x_{3}0\\ g_{3}\left(\vec{x}\right)=-\pi x_{3}^{2}x_{4}-\frac{4}{3}\pi x_{3}^{3}+12,960,000\\ g_{4}\left(\vec{x}\right)=x_{4}-2400 \end{array} \end{array}$$

Parameters range: $0⩽x_{1},x_{2}⩽99,10⩽x_{3},x_{4}⩽200.$

**Figure 13:**
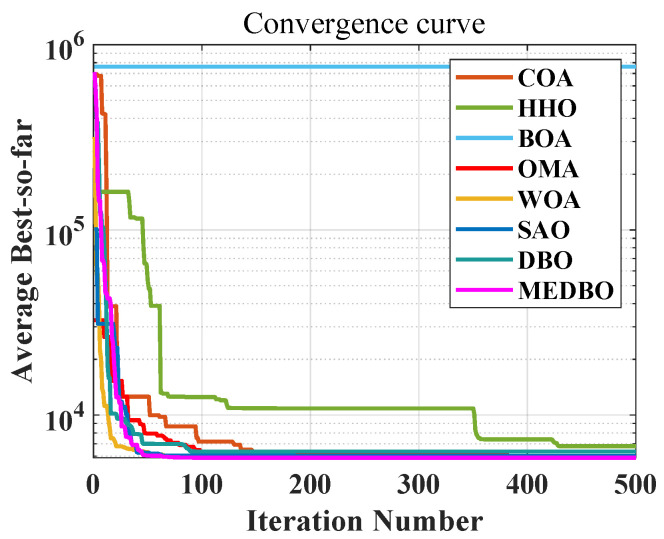
The iteration plot of the pressure vessel problem.

A comprehensive examination of both the pressure vessel model depicted in Figure 13 and the iteration plot for the algorithm addressing the pressure-vessel-design problem, as depicted in Figure 13, highlights the MEDBO algorithm’s exceptional efficiency in achieving the optimal solution with the fewest iterations. These results not only validate the effectiveness and reliability of the MEDBO algorithm in tackling real-world engineering challenges but also offer valuable insights for further research and practical applications in the field of pressure vessel design.

Furthermore, the iteration plot in Figure 13 demonstrates the superior performance of the MEDBO algorithm compared to all other algorithms. MEDBO consistently outperforms other algorithms when addressing the same problem, further proving its potential for application in pressure vessel design. By comparing the performance of different algorithms, we can gain a deeper understanding of their strengths and weaknesses, thus providing robust support for practical engineering applications.

In Table 11, we present a comprehensive statistical analysis derived from 100 repeated experiments conducted on the pressure-vessel-design problem. Notably, among the eight algorithms scrutinized, MEDBO demonstrates the lowest mean value. Although it exhibits slightly less stability compared to the WOA algorithm, MEDBO still showcases stronger stability than DBO. This suggests that, when appropriately configured, MEDBO offers significant advantages in tackling the pressure-vessel-design problem. Across both mean and stability metrics, MEDBO outperforms other algorithms, underscoring its superiority. These findings not only highlight the effectiveness of MEDBO in addressing the pressure-vessel-design problem but also offer robust support for further research and practical implementation.

## 6. Conclusions

This paper introduces an enhanced algorithm, MEDBO, which incorporates novel strategies inspired by the natural behaviors of rolling and foraging dung beetles. By implementing a lateral movement strategy and an intelligent evasion mechanism, MEDBO significantly improves the algorithm’s search scope and adaptability. These enhancements aim to overcome the initial shortcomings of the original DBO algorithm, resulting in more efficient and accurate optimization solutions. To evaluate the performance of MEDBO, we utilized the CEC2017 benchmark functions and compared it with other classical algorithms. MEDBO ranked first in several functions. Additionally, we tested three engineering applications: the tension (compression) design problem, the three-bar truss-design problem, and the pressure vessel problem. The results demonstrated that MEDBO performs exceptionally well and holds practical application value. Through these tests and applications, MEDBO not only showcased superior performance in theoretical benchmarks but also proved its effectiveness and practicality in real-world engineering problems, indicating its broad potential and applicability in solving complex optimization issues.

Looking ahead, future research can explore and enhance MEDBO in several ways. Firstly, further optimization of MEDBO’s parameter selection and tuning strategies could improve its performance on larger and more complex problems. Secondly, hybrid algorithms that combine the strengths of other swarm intelligence algorithms could be developed to further enhance optimization efficiency and accuracy. Additionally, exploring MEDBO’s application to a wider range of real-world engineering problems could validate its applicability and effectiveness across different domains.

## Figures and Tables

**Table 1:** CEC2017 functions.

Type	No.	Function	Minimum Value
Unimodal functions	1	Shifted and Rotated Bent Cigar Function	100
	2	Shifted and Rotated Zakharov Function	200
			
Simple multimodal functions	3	Shifted and Rotated Rosenbrock’s Function	300
	4	Shifted and Rotated Rastrigin’s Function	400
	5	Shifted and Rotated Expanded Scaffer’s F6 Function	500
	6	Shifted and Rotated Lunacek Bi_Rastrigin Function	600
	7	Shifted and Rotated Noncontinuous Rastrigin’s Function	700
	8	Shifted and Rotated Levy Function	800
	9	Shifted and Rotated Schwefel’s Function	900
			
Hybrid functions	10	Hybrid Function 1 (N = 3)	1000
	11	Hybrid function 2 (N = 3)	1100
	12	Hybrid function 3 (N = 3)	1200
	13	Hybrid function 4 (N = 4)	1300
	14	Hybrid function 5 (N = 4)	1400
	15	Hybrid function 6 (N = 4)	1500
	16	Hybrid function 6 (N = 5)	1600
	17	Hybrid function 6 (N = 5)	1700
	18	Hybrid function 6 (N = 5)	1800
	19	Hybrid function 6 (N = 6)	1900
			
Composition functions	20	Composition Function 1 (N = 3)	2000
	21	Composition function 2 (N = 3)	2100
	22	Composition function 3 (N = 4)	2200
	23	Composition function 4 (N = 4)	2300
	24	Composition function 5 (N = 5)	2400
	25	Composition function 6 (N = 5)	2500
	26	Composition function 7 (N = 6)	2600
	27	Composition function 7 (N = 6)	2700
	28	Composition function 9 (N = 3)	2800
	29	Composition function 10 (N = 3)	2900
Search range: ${\left[-100,100\right]}^{D}$			

**Table 2:** Algorithm parameters.

Algorithm	Population	Number of Iterations	Parameters
COA [35]	30	500	temp =15∗rand+20
HHO [3]	30	500	β=1.5;r=0.5;E=0.5
BOA [36]	30	500	P=0.8;pe=0.1;sm=0.01
OMA [37]	30	500	NA=1.40
WOA [38]	30	500	$a=2*(1-t/T_{\max});k=1$
SAO [39]	30	500	T=0;
DBO [5]	30	500	RDB=6;EDB=6;FDB=7;SDB=11
MEDBO	30	500	$v_{o}=0;r_{o}=1;g=0.980665$

**Table 3:** CEC2017 test results for 30 dimensions.

		Dim = 30							
		**COA**	**HHO**	**BOA**	**OMA**	**WOA**	**SAO**	**DBO**	**MEDBO**
F1	min	9.88E+06	1.26E+08	4.88E+10	3.93E+08	4.25E+05	1.31E+07	8.27E+05	1.37E+04
	mean	5.76E+08	4.24E+08	7.75E+10	1.56E+09	2.25E+08	**2.16E+07**	2.67E+08	7.14E+07
	std	3.39E+17	6.59E+16	8.09E+19	9.65E+17	3.52E+17	1.61E+19	4.07E+16	8.16E+15
	degree	5	4	8	6	2	1	3	2
F2	min	6.28E+04	4.24E+04	8.78E+04	3.79E+04	5.86E+03	5.27E+04	7.19E+04	6.70E+04
	mean	1.04E+05	5.72E+04	1.18E+07	6.73E+04	**1.25E+04**	8.72E+04	9.76E+04	9.68E+04
	std	7.18E+08	8.59E+07	3.67E+15	2.08E+08	2.53E+07	2.60E+08	1.36E+09	6.53E+08
	degree	7	2	8	3	1	4	6	5
F3	min	5.04E+02	6.16E+02	3.77E+03	5.73E+02	4.89E+02	1.56E+03	5.15E+02	4.88E+02
	mean	6.03E+02	7.39E+02	1.68E+04	8.26E+02	5.61E+02	3.18E+03	6.67E+02	**5.58E+02**
	std	7.86E+03	1.97E+04	3.62E+07	3.33E+04	1.93E+03	1.13E+06	2.02E+04	2.34E+03
	degree	3	5	8	6	2	7	4	1
F4	min	5.62E+02	6.87E+02	9.06E+02	6.49E+02	6.41E+02	7.66E+02	7.00E+02	5.51E+02
	mean	7.50E+02	7.69E+02	1.01E+03	7.18E+02	6.94E+02	8.26E+02	7.81E+02	**5.86E+02**
	std	3.71E+03	1.12E+03	3.21E+03	1.11E+03	1.07E+03	7.09E+02	2.14E+03	2.97E+03
	degree	4	5	8	3	2	7	6	1
F5	min	6.20E+02	6.58E+02	6.94E+02	6.10E+02	6.34E+02	6.50E+02	6.19E+02	6.30E+02
	mean	6.51E+02	6.66E+02	7.09E+02	6.29E+02	6.47E+02	**6.17E+02**	6.46E+02	6.31E+02
	std	1.79E+02	2.76E+01	1.48E+02	9.18E+01	5.64E+01	4.91E+01	1.53E+02	1.11E+02
	degree	6	7	8	2	5	1	4	3
F6	min	9.80E+02	1.22E+03	1.33E+03	1.04E+03	9.73E+02	1.14E+03	8.60E+02	8.81E+02
	mean	1.22E+03	1.33E+03	1.50E+03	1.19E+03	1.08E+03	1.26E+03	1.00E+03	**9.4E0+02**
	std	1.06E+04	3.00E+03	6.98E+03	6.82E+03	3.25E+03	3.87E+03	7.58E+03	8.21E+03
	degree	5	7	8	4	3	6	2	1
F7	min	9.10E+02	9.37E+02	1.13E+03	9.13E+02	8.91E+02	1.03E+03	9.21E+02	7.00E+02
	mean	9.78E+02	9.86E+02	1.21E+03	9.76E+02	9.41E+02	1.10E+03	1.02E+03	**7.02E+02**
	std	5.96E+02	8.69E+02	1.91E+03	1.66E+03	5.66E+02	9.20E+02	3.27E+03	2.91E+03
	degree	4	5	8	3	2	7	6	1
F8	min	3.75E+03	5.35E+03	1.17E+04	2.29E+03	2.21E+03	1.24E+03	3.40E+03	3.02E+03
	mean	7.64E+03	8.22E+03	1.64E+04	3.85E+03	3.97E+03	**1.60E+03**	6.95E+03	7.38E+03
	std	3.12E+06	1.22E+06	5.37E+06	2.12E+06	7.61E+05	3.57E+06	4.80E+06	8.22E+06
	degree	6	7	8	2	3	1	4	5
F9	min	4.22E+03	4.82E+03	8.90E+03	6.32E+03	3.64E+03	8.00E+03	5.04E+03	4.03E+03
	mean	6.24E+03	6.11E+03	1.03E+04	8.47E+03	4.98E+03	8.97E+03	6.67E+03	**4.63E+03**
	std	9.43E+05	5.07E+05	2.61E+05	3.39E+05	3.69E+05	1.03E+05	1.12E+06	5.36E+05
	degree	4	3	8	6	2	7	5	1
F10	min	1.40E+03	1.27E+03	6.82E+03	1.26E+03	1.19E+03	2.01E+03	1.25E+03	1.27E+03
	mean	1.88E+03	1.56E+03	2.71E+04	1.40E+03	**1.27E+03**	4.02E+03	1.87E+03	1.52E+03
	std	2.92E+05	3.37E+04	2.90E+08	7.80E+03	2.52E+03	1.09E+06	2.80E+05	3.15E+04
	degree	6	4	8	2	1	7	5	3
F11	min	2.39E+06	1.24E+07	1.31E+10	7.52E+05	1.24E+05	1.87E+09	1.12E+06	1.54E+06
	mean	1.80E+07	7.32E+07	2.22E+10	1.89E+07	2.76E+06	3.04E+09	7.70E+07	**1.68E+06**
	std	2.07E+14	5.54E+15	2.45E+19	4.11E+14	1.92E+13	8.28E+17	1.93E+16	5.86E+13
	degree	3	5	8	4	2	7	6	1
F12	min	4.25E+04	1.40E+05	9.76E+09	1.17E+04	4.34E+03	4.15E+08	1.57E+04	9.29E+03
	mean	3.14E+05	1.26E+06	2.06E+10	1.48E+05	**1.30E+04**	1.35E+09	8.77E+06	4.61E+04
	std	2.54E+11	1.07E+12	4.51E+19	2.97E+11	1.03E+08	4.78E+17	2.90E+14	1.46E+09
	degree	4	5	8	3	1	7	6	2
F13	min	4.94E+03	6.62E+03	2.55E+06	2.18E+03	1.59E+03	1.06E+05	1.18E+04	1.26E+04
	mean	3.62E+05	1.20E+06	3.32E+07	2.03E+04	**4.09E+03**	8.32E+05	3.01E+05	2.17E+05
	std	1.43E+11	2.26E+12	1.53E+15	9.68E+08	1.32E+07	5.68E+11	2.00E+11	4.82E+10
	degree	5	7	8	2	1	6	4	3
F14	min	6.69E+03	3.22E+04	3.74E+08	2.74E+03	1.73E+03	1.05E+06	2.36E+03	5.48E+03
	mean	2.34E+04	1.28E+05	2.93E+09	8.70E+03	**6.40E+03**	6.32E+07	1.18E+05	3.10E+04
	std	4.94E+08	8.35E+09	2.42E+18	2.79E+07	5.87E+07	3.09E+15	5.10E+10	5.52E+08
	degree	3	6	8	2	1	7	5	4
F15	min	2.30E+03	2.46E+03	4.50E+03	2.79E+03	2.28E+03	3.58E+03	2.56E+03	2.39E+03
	mean	3.10E+03	3.61E+03	5.71E+03	3.34E+03	2.79E+03	4.25E+03	3.30E+03	**2.52E+03**
	std	1.68E+05	1.74E+05	1.37E+06	5.64E+04	6.94E+04	6.36E+04	2.24E+05	1.05E+05
	degree	3	6	8	5	2	7	4	1
F16	min	1.90E+03	2.38E+03	3.17E+03	1.92E+03	1.88E+03	2.44E+03	2.06E+03	2.18E+03
	mean	2.36E+03	2.71E+03	4.61E+03	2.21E+03	2.38E+03	2.85E+03	2.62E+03	**2.20E+03**
	std	4.91E+04	5.14E+04	9.64E+06	2.04E+04	6.19E+04	3.52E+04	5.79E+04	7.21E+04
	degree	3	6	8	2	4	7	5	1
F17	min	9.72E+04	2.69E+05	2.37E+07	8.70E+04	2.45E+04	3.24E+06	1.84E+05	1.02E+05
	mean	3.77E+06	3.48E+06	3.41E+08	4.54E+05	1.26E+05	1.47E+07	4.27E+06	**1.24E+06**
	std	1.57E+13	9.47E+12	8.63E+16	2.35E+11	8.40E+09	8.72E+13	3.95E+13	5.64E+12
	degree	5	4	8	3	3	7	6	1
F18	min	3.54E+03	1.63E+05	3.59E+08	2.07E+03	1.95E+03	6.33E+06	2.63E+03	3.30E+03
	mean	3.78E+04	2.14E+06	3.69E+09	1.42E+04	**1.08E+04**	8.57E+07	3.46E+06	4.79E+04
	std	2.94E+09	4.33E+12	3.34E+18	1.05E+08	1.71E+08	1.92E+15	3.37E+13	5.51E+09
	degree	3	5	8	2	1	7	6	4
F19	min	2.28E+03	2.38E+03	3.10E+03	2.43E+03	2.20E+03	1.90E+03	2.18E+03	2.30E+03
	mean	2.74E+03	2.84E+03	3.63E+03	2.66E+03	2.55E+03	**1.97E+03**	2.73E+03	2.47E+03
	std	4.11E+04	3.40E+04	5.04E+04	2.15E+04	3.27E+04	3.18E+04	4.55E+04	6.53E+04
	degree	6	7	8	4	3	1	5	2
F20	min	2.41E+03	2.47E+03	2.64E+03	2.41E+03	2.42E+03	2.56E+03	2.44E+03	2.04E+03
	mean	2.49E+03	2.59E+03	2.76E+03	2.47E+03	2.47E+03	2.60E+03	2.55E+03	**2.11E+03**
	std	2.25E+03	3.78E+03	3.91E+03	8.46E+02	1.36E+03	6.72E+02	2.34E+03	2.35E+03
	degree	4	6	8	2	3	7	5	1
F21	min	2.33E+03	2.61E+03	6.89E+03	2.47E+03	2.32E+03	2.57E+03	2.36E+03	2.33E+03
	mean	3.49E+03	7.09E+03	1.10E+04	**3.11E+03**	3.89E+03	3.12E+03	4.99E+03	5.70E+03
	std	4.60E+06	3.04E+06	1.90E+06	1.98E+06	2.75E+06	1.82E+06	5.61E+06	4.32E+06
	degree	3	7	8	1	4	2	5	6
F22	min	2.75E+03	3.02E+03	3.15E+03	2.80E+03	2.82E+03	3.01E+03	2.79E+03	2.43E+03
	mean	2.89E+03	3.30E+03	3.47E+03	2.89E+03	2.94E+03	3.07E+03	3.01E+03	**2.48E+03**
	std	5.23E+03	2.73E+04	3.97E+04	1.48E+03	7.04E+03	1.55E+03	9.30E+03	5.34E+03
	degree	2	7	8	3	4	6	5	1
F23	min	2.91E+03	3.23E+03	3.23E+03	3.02E+03	2.97E+03	3.16E+03	3.06E+03	2.67E+03
	mean	3.03E+03	3.56E+03	3.51E+03	3.12E+03	3.09E+03	3.26E+03	3.19E+03	**2.77E+03**
	std	6.51E+03	3.40E+04	5.53E+04	5.49E+03	4.35E+03	1.92E+03	6.45E+03	5.90E+03
	degree	2	8	7	4	3	6	5	1
F24	min	2.91E+03	2.96E+03	4.88E+03	2.97E+03	2.91E+03	3.19E+03	2.89E+03	2.89E+03
	mean	2.98E+03	3.02E+03	5.74E+03	3.07E+03	2.95E+03	3.59E+03	2.99E+03	**2.93E+03**
	std	1.80E+03	1.45E+03	4.07E+05	3.89E+03	7.45E+02	5.81E+04	2.84E+03	1.01E+03
	degree	3	5	8	6	2	7	4	1
F25	min	3.45E+03	3.79E+03	8.19E+03	5.25E+03	5.54E+03	7.00E+03	4.01E+03	2.92E+03
	mean	6.31E+03	8.07E+03	1.14E+04	6.46E+03	7.11E+03	7.93E+03	7.10E+03	6.78E+03
	std	1.81E+06	1.87E+06	1.67E+06	3.70E+05	1.05E+06	2.28E+05	6.55E+05	2.20E+06
	degree	1	7	8	2	5	6	4	3
F26	min	3.24E+03	3.35E+03	3.54E+03	3.27E+03	3.22E+03	3.45E+03	3.23E+03	2.93E+03
	mean	3.29E+03	3.65E+03	4.09E+03	3.31E+03	3.36E+03	3.57E+03	3.33E+03	**2.98E+03**
	std	1.68E+03	3.60E+04	2.00E+05	8.84E+02	4.78E+03	5.85E+03	3.73E+03	2.95E+03
	degree	2	7	8	3	5	6	4	1
F27	min	3.28E+03	3.36E+03	5.24E+03	3.36E+03	3.22E+03	3.98E+03	3.28E+03	3.28E+03
	mean	3.41E+03	3.51E+03	7.83E+03	3.60E+03	**3.32E+03**	4.40E+03	3.83E+03	3.44E+03
	std	1.90E+04	1.19E+04	1.15E+06	4.37E+04	2.15E+03	9.83E+04	8.24E+05	1.48E+05
	degree	2	4	8	5	1	7	6	3
F28	min	3.66E+03	4.25E+03	5.47E+03	3.91E+03	3.84E+03	4.50E+03	3.71E+03	3.59E+03
	mean	4.21E+03	5.13E+03	8.30E+03	**4.16E+03**	4.40E+03	5.13E+03	4.54E+03	4.35E+03
	std	7.34E+04	3.31E+05	2.19E+07	2.32E+04	1.06E+05	1.14E+05	1.23E+05	1.10E+05
	degree	2	7	8	1	4	6	5	3
F29	min	1.10E+05	1.94E+06	4.66E+08	3.82E+04	7.50E+03	6.40E+07	7.80E+03	1.30E+04
	mean	1.13E+06	1.52E+07	2.98E+09	2.70E+05	**3.08E+04**	2.18E+08	6.60E+06	2.55E+05
	std	1.30E+12	2.00E+14	2.54E+18	5.59E+10	1.55E+09	5.93E+15	1.15E+14	1.60E+11
	degree	4	6	8	3	1	7	5	2

**Table 4:** CEC2017 test results for 100 dimensions.

		Dim = 100							
		**COA**	**HHO**	**BOA**	**OMA**	**WOA**	**SAO**	**DBO**	**MEDBO**
F1	min	4.82E+10	3.98E+10	2.67E+11	7.95E+10	3.94E+10	1.86E+10	2.45E+10	2.66E+10
	mean	6.82E+10	5.24E+10	2.93E+11	1.14E+11	6.51E+10	**2.11E+11**	1.01E+11	2.74E+10
	std	1.14E+20	5.25E+19	5.00E+19	2.25E+20	1.86E+20	1.74E+20	5.07E+21	3.08E+21
	degree	5	3	8	7	4	1	6	2
F2	min	4.85E+05	3.26E+05	4.12E+05	3.29E+05	2.04E+05	4.17E+05	3.47E+05	3.27E+05
	mean	7.29E+05	3.46E+05	4.28E+10	4.25E+05	**2.40E+05**	5.80E+05	7.06E+05	7.03E+05
	std	1.77E+10	1.30E+08	2.41E+22	2.18E+09	3.15E+08	1.00E+10	6.89E+10	6.90E+10
	degree	7	2	8	3	1	4	6	5
F3	min	5.39E+03	6.73E+03	9.16E+04	1.08E+04	3.47E+03	2.36E+03	3.62E+03	2.27E+03
	mean	9.39E+03	9.67E+03	1.16E+05	1.60E+04	7.07E+03	**3.18E+03**	2.01E+04	3.46E+03
	std	6.18E+06	3.63E+06	2.20E+08	1.23E+07	6.16E+06	1.04E+08	2.37E+08	1.27E+08
	degree	4	5	8	6	3	1	7	2
F4	min	1.47E+03	1.57E+03	2.13E+03	1.46E+03	1.35E+03	1.89E+03	1.37E+03	9.38E+02
	mean	1.54E+03	1.69E+03	2.26E+03	1.68E+03	1.44E+03	2.06E+03	1.65E+03	**1.24E+03**
	std	3.00E+03	4.99E+03	3.97E+03	1.91E+04	3.11E+03	5.55E+03	4.71E+04	3.82E+04
	degree	3	6	8	5	2	7	4	1
F5	min	6.65E+02	6.82E+02	7.14E+02	6.71E+02	6.58E+02	6.24E+02	6.60E+02	6.20E+02
	mean	6.74E+02	6.91E+02	7.25E+02	6.85E+02	6.70E+02	7.06E+02	6.78E+02	**6.35E+02**
	std	1.05E+01	1.64E+01	2.66E+01	5.50E+01	2.60E+01	2.65E+01	2.08E+02	1.22E+02
	degree	3	6	8	5	2	7	4	1
F6	min	2.83E+03	3.51E+03	3.95E+03	3.27E+03	2.82E+03	2.76E+03	2.56E+03	2.68E+03
	mean	3.38E+03	3.75E+03	4.22E+03	4.14E+03	3.19E+03	**2.89E+03**	2.95E+03	3.09E+03
	std	3.10E+04	9.68E+03	1.34E+04	1.93E+05	2.69E+04	5.57E+04	6.09E+04	4.01E+04
	degree	5	6	8	7	4	1	2	3
F7	min	1.87E+03	2.00E+03	2.58E+03	1.76E+03	1.73E+03	2.26E+03	1.74E+03	1.70E+03
	mean	2.01E+03	2.11E+03	2.76E+03	2.01E+03	**1.90E+03**	2.44E+03	2.13E+03	2.16E+03
	std	2.70E+03	4.52E+03	7.54E+03	1.65E+04	5.15E+03	5.94E+03	4.05E+04	4.91E+04
	degree	2	4	8	3	1	7	5	6
F8	min	3.28E+04	6.17E+04	9.21E+04	5.83E+04	2.49E+04	7.02E+04	6.21E+04	2.90E+04
	mean	4.49E+04	6.89E+04	1.09E+05	7.23E+04	3.09E+04	9.28E+04	7.97E+04	**3.00E+04**
	std	1.11E+08	1.51E+07	5.71E+07	6.05E+07	2.28E+07	7.76E+07	7.31E+07	1.00E+08
	degree	3	4	8	5	2	7	6	1
F9	min	1.88E+04	2.18E+04	3.40E+04	2.97E+04	1.63E+04	3.18E+04	2.03E+04	1.77E+04
	mean	2.38E+04	2.44E+04	3.54E+04	3.21E+04	1.95E+04	3.33E+04	2.84E+04	**1.86E+04**
	std	1.08E+07	2.58E+06	6.21E+05	5.80E+05	2.81E+06	3.21E+05	1.85E+07	9.90E+06
	degree	3	4	8	6	2	7	5	1
F10	min	1.45E+05	8.16E+04	4.66E+05	7.07E+04	1.84E+04	1.43E+05	1.40E+05	5.84E+04
	mean	3.30E+05	1.45E+05	1.48E+07	9.94E+04	**3.39E+04**	1.79E+05	2.19E+05	1.99E+05
	std	1.32E+10	1.13E+09	2.55E+15	3.08E+08	9.86E+07	5.92E+08	2.56E+09	6.09E+09
	degree	7	3	8	2	1	4	6	5
F11	min	5.26E+09	4.85E+09	2.05E+11	9.78E+09	1.34E+09	1.56E+08	4.13E+09	1.84E+09
	mean	1.39E+10	1.02E+10	2.50E+11	2.08E+10	7.25E+09	**4.42E+08**	7.68E+09	5.20E+09
	std	4.88E+19	8.42E+18	1.88E+20	4.72E+19	3.10E+19	2.21E+20	4.79E+18	6.36E+18
	degree	6	5	8	7	3	1	4	2
F12	min	1.17E+07	6.21E+07	5.03E+10	3.58E+08	3.63E+05	1.34E+04	2.62E+07	8.02E+06
	mean	3.56E+08	2.59E+08	6.13E+10	1.28E+09	1.90E+08	**6.78E+05**	2.88E+08	9.63E+07
	std	3.57E+17	2.54E+16	2.16E+19	1.01E+18	1.59E+17	6.39E+18	3.58E+16	1.15E+16
	degree	6	4	8	7	3	1	5	2
F13	min	2.65E+06	3.47E+06	2.18E+08	1.02E+06	3.77E+05	3.36E+05	4.20E+06	2.35E+06
	mean	1.02E+07	9.87E+06	4.94E+08	4.73E+06	2.00E+06	**9.01E+05**	2.05E+07	1.29E+07
	std	3.27E+13	1.97E+13	6.45E+16	6.27E+12	2.03E+12	1.01E+15	1.11E+14	1.22E+14
	degree	5	4	8	3	2	1	7	6
F14	min	2.50E+05	4.80E+06	2.72E+10	3.86E+06	1.34E+04	9.61E+03	1.42E+06	1.55E+04
	mean	1.17E+08	1.50E+07	3.65E+10	3.76E+07	1.78E+06	**1.32E+03**	7.88E+07	2.19E+04
	std	1.90E+17	7.01E+13	2.20E+19	1.31E+15	4.87E+13	5.03E+18	5.79E+15	7.68E+14
	degree	7	4	8	5	3	1	6	2
F15	min	6.68E+03	8.43E+03	1.75E+04	8.18E+03	5.59E+03	5.28E+03	7.42E+03	6.56E+03
	mean	8.94E+03	1.04E+04	2.32E+04	9.94E+03	7.61E+03	7.55E+03	9.13E+03	**6.96E+03**
	std	2.20E+06	1.14E+06	7.38E+06	1.72E+06	8.01E+05	1.17E+06	1.40E+06	1.59E+06
	degree	4	7	8	6	3	2	5	1
F16	min	5.09E+03	5.80E+03	1.13E+06	5.07E+03	4.89E+03	1.20E+04	7.51E+03	3.15E+03
	mean	7.14E+03	8.26E+03	2.54E+07	6.72E+03	7.39E+03	8.63E+04	9.83E+03	**4.24E+03**
	std	1.14E+06	4.13E+06	4.30E+14	9.28E+05	2.02E+06	4.12E+09	2.18E+06	1.31E+06
	degree	3	5	8	2	4	7	6	1
F17	min	2.14E+06	3.46E+06	2.95E+08	2.29E+06	1.09E+06	4.83E+07	2.81E+06	1.15E+06
	mean	9.10E+06	1.05E+07	8.54E+08	5.38E+06	2.89E+06	1.31E+08	2.60E+07	**1.98E+06**
	std	2.96E+13	2.03E+13	1.07E+17	6.16E+12	1.75E+12	4.19E+15	3.23E+14	1.32E+14
	degree	4	5	8	3	2	7	6	1
F18	min	2.72E+06	9.21E+06	2.10E+10	1.32E+07	4.50E+04	2.62E+04	2.00E+07	2.04E+06
	mean	2.31E+07	3.01E+07	3.73E+10	7.64E+07	1.79E+06	**1.69E+05**	8.93E+07	3.72E+07
	std	6.60E+14	2.41E+14	3.12E+19	4.35E+15	1.48E+13	1.56E+18	8.44E+15	3.57E+15
	degree	3	4	8	6	2	1	7	5
F19	min	5.32E+03	5.26E+03	8.02E+03	6.98E+03	4.55E+03	7.13E+03	5.70E+03	5.01E+03
	mean	7.04E+03	6.07E+03	9.07E+03	7.46E+03	5.42E+03	7.98E+03	7.20E+03	**5.41E+03**
	std	3.74E+05	2.46E+05	1.86E+05	6.76E+04	2.28E+05	1.16E+05	4.02E+05	5.56E+05
	degree	4	3	8	6	2	7	5	1
F20	min	3.49E+03	3.85E+03	4.41E+03	3.28E+03	3.33E+03	4.02E+03	3.74E+03	2.72E+03
	mean	3.79E+03	4.42E+03	4.91E+03	3.44E+03	3.59E+03	4.22E+03	4.02E+03	**3.04E+03**
	std	3.80E+04	5.27E+04	5.19E+04	6.08E+03	1.52E+04	9.04E+03	3.25E+04	4.21E+04
	degree	4	7	8	2	3	6	5	1
F21	min	2.35E+04	2.52E+04	3.53E+04	3.29E+04	2.13E+04	3.27E+04	2.13E+04	2.00E+04
	mean	2.97E+04	2.73E+04	3.77E+04	3.48E+04	2.33E+04	3.52E+04	2.96E+04	**2.31E+04**
	std	1.00E+07	1.69E+06	1.20E+06	3.88E+05	1.18E+06	4.96E+05	2.48E+07	1.26E+07
	degree	5	3	8	6	2	7	4	1
F22	min	3.93E+03	5.04E+03	5.30E+03	3.92E+03	4.10E+03	5.10E+03	4.32E+03	3.24E+03
	mean	4.30E+03	5.81E+03	6.17E+03	4.17E+03	4.62E+03	5.29E+03	4.78E+03	**3.72E+03**
	std	3.78E+04	1.09E+05	3.62E+05	2.18E+04	5.46E+04	1.17E+04	5.81E+04	6.41E+04
	degree	3	7	8	2	4	6	5	1
F23	min	4.78E+03	6.66E+03	7.70E+03	5.29E+03	5.14E+03	5.11E+03	4.86E+03	4.71E+03
	mean	5.40E+03	8.12E+03	1.01E+04	5.78E+03	6.08E+03	5.33E+03	6.04E+03	**5.31E+03**
	std	1.02E+05	4.49E+05	4.46E+06	1.06E+05	1.73E+05	9.88E+04	2.91E+05	9.57E+04
	degree	3	7	8	4	6	2	5	1
F24	min	5.88E+03	5.84E+03	2.71E+04	8.50E+03	5.20E+03	4.74E+03	4.83E+03	4.82E+03
	mean	8.13E+03	6.81E+03	3.15E+04	1.23E+04	7.42E+03	**5.16E+03**	9.23E+03	5.78E+03
	std	1.62E+06	3.22E+05	5.58E+06	5.70E+06	1.15E+06	5.68E+06	3.10E+07	5.54E+07
	degree	5	3	8	7	4	1	6	2
F25	min	2.41E+04	2.87E+04	4.96E+04	3.01E+04	2.62E+04	1.89E+04	2.05E+04	1.80E+04
	mean	3.23E+04	3.17E+04	5.94E+04	3.63E+04	3.14E+04	**2.09E+04**	2.71E+04	2.18E+04
	std	1.27E+07	2.98E+06	1.52E+07	1.30E+07	5.82E+06	5.91E+06	1.52E+07	8.69E+06
	degree	6	5	8	7	4	1	3	2
F26	min	4.08E+03	5.29E+03	8.22E+03	4.81E+03	4.45E+03	3.43E+03	4.00E+03	3.41E+03
	mean	4.47E+03	6.89E+03	1.11E+04	5.52E+03	5.21E+03	8.44E+03	4.69E+03	**4.30E+03**
	std	4.25E+04	6.77E+05	2.32E+06	2.34E+05	3.30E+05	2.97E+05	2.18E+05	6.23E+04
	degree	2	6	8	5	4	7	3	1
F27	min	8.40E+03	8.13E+03	3.24E+04	1.25E+04	6.48E+03	1.13E+04	7.40E+03	8.17E+03
	mean	1.20E+04	9.43E+03	3.85E+04	1.44E+04	9.13E+03	**1.27E+04**	1.82E+04	1.85E+04
	std	3.43E+06	5.23E+05	6.99E+06	1.17E+06	1.67E+06	5.44E+06	4.84E+07	3.36E+07
	degree	4	3	8	5	2	1	6	7
F28	min	9.39E+03	1.10E+04	1.39E+05	1.10E+04	8.66E+03	2.20E+04	8.68E+03	7.92E+03
	mean	1.13E+04	1.29E+04	2.04E+06	1.33E+04	1.11E+04	3.53E+04	1.26E+04	**1.06E+04**
	std	1.43E+06	1.78E+06	3.71E+12	5.88E+06	2.45E+06	1.09E+08	7.81E+06	2.02E+06
	degree	3	5	8	6	2	7	4	1
F29	min	3.78E+07	1.90E+08	3.80E+10	2.11E+08	8.96E+06	9.47E+05	9.56E+07	1.82E+07
	mean	8.22E+08	7.91E+08	5.71E+10	1.14E+09	5.25E+08	**9.89E+05**	3.27E+08	7.97E+07
	std	1.78E+18	1.64E+17	3.63E+19	1.42E+18	1.46E+18	7.77E+18	3.37E+16	2.40E+15
	degree	6	5	8	7	4	1	3	2

**Table 5:** Wilcoxon rank-sum test (Dim = 30).

	SSA	HHO	BOA	OMA	WOA	SCA	DBO
F1	1.61E-10	9.92E-11	3.02E-11	3.02E-11	1.29E-06	3.02E-11	7.77E-09
F2	1.78E-10	6.15E-02	3.02E-11	1.91E-01	3.02E-11	5.87E-04	9.92E-11
F3	5.08E-03	5.97E-09	3.02E-11	2.15E-10	3.40E-01	3.52E-07	2.43E-05
F4	1.50E-02	5.53E-08	3.02E-11	2.90E-01	1.54E-01	8.56E-04	4.64E-03
F5	3.50E-03	1.96E-10	3.02E-11	4.69E-08	5.01E-02	1.71E-01	2.42E-02
F6	1.20E-08	7.39E-11	3.02E-11	5.19E-07	2.81E-02	5.60E-07	3.67E-03
F7	9.05E-02	2.07E-02	3.02E-11	4.20E-01	6.91E-04	2.84E-01	6.74E-06
F8	1.25E-04	2.83E-08	3.34E-11	2.60E-05	2.15E-06	8.42E-01	1.41E-01
F9	2.42E-02	6.52E-01	3.34E-11	4.20E-10	3.59E-05	3.59E-05	4.51E-02
F10	7.38E-10	3.81E-07	3.02E-11	4.55E-01	3.01E-04	3.67E-03	2.68E-06
F11	2.50E-03	8.15E-11	3.02E-11	3.83E-05	4.03E-03	5.97E-05	1.86E-06
F12	2.89E-03	7.22E-06	3.02E-11	7.24E-02	1.43E-08	3.37E-04	2.00E-06
F13	7.96E-01	7.20E-05	3.02E-11	2.44E-09	3.02E-11	8.20E-07	6.63E-01
F14	5.32E-03	4.98E-11	3.02E-11	7.51E-01	2.92E-02	6.95E-01	6.52E-09
F15	9.23E-01	1.49E-06	3.02E-11	1.49E-04	5.55E-02	1.76E-01	3.77E-04
F16	1.44E-03	1.54E-01	3.02E-11	4.94E-05	2.92E-02	6.20E-01	1.22E-01
F17	5.87E-04	1.19E-06	3.02E-11	5.75E-02	1.16E-07	9.23E-01	7.70E-04
F18	1.17E-04	3.02E-11	3.02E-11	7.29E-03	8.65E-01	2.77E-01	8.10E-10
F19	9.23E-01	5.37E-02	3.02E-11	6.57E-02	1.24E-03	8.88E-01	3.27E-02
F20	9.33E-02	6.05E-07	3.34E-11	2.40E-01	4.84E-02	9.82E-01	4.64E-05
F2 1	3.27E-02	5.53E-08	4.50E-11	1.70E-02	3.87E-01	3.27E-02	4.64E-03
F22	2.92E-02	4.98E-11	3.02E-11	2.23E-01	8.24E-02	6.95E-01	1.17E-03
F23	5.27E-05	8.99E-11	6.70E-11	7.28E-01	3.79E-01	3.78E-02	4.03E-03
F24	5.30E-01	1.87E-07	3.02E-11	8.15E-11	5.19E-02	4.50E-11	9.07E-03
F25	4.20E-01	2.20E-07	3.02E-11	3.95E-01	6.15E-02	1.91E-01	4.46E-04
F26	1.41E-01	2.15E-10	3.02E-11	8.68E-03	2.16E-03	1.30E-03	2.32E-02
F27	9.47E-03	3.16E-10	3.02E-11	6.72E-10	5.01E-02	4.98E-11	1.58E-04
F28	7.73E-02	1.43E-05	3.69E-11	1.44E-02	7.62E-01	1.56E-02	8.19E-01
F29	9.79E-05	1.78E-10	3.02E-11	7.39E-01	4.69E-08	2.44E-09	5.57E-03

**Table 6:** Wilcoxon rank-sum test (Dim = 100).

	SSA	HHO	BOA	OMA	WOA	SCA	DBO
F1	1.69E-09	1.68E-04	2.97E-11	3.02E-11	1.87E-07	3.02E-11	2.90E-01
F2	3.02E-11	8.20E-07	5.49E-11	7.62E-03	3.02E-11	1.10E-08	8.48E-09
F3	1.61E-10	6.70E-11	3.02E-11	3.02E-11	4.57E-09	3.02E-11	5.07E-10
F4	9.07E-03	4.74E-06	3.02E-11	1.44E-03	1.49E-06	3.69E-11	6.41E-01
F5	1.99E-02	4.20E-10	3.02E-11	5.27E-05	1.95E-03	6.70E-11	4.04E-01
F6	5.60E-07	3.02E-11	3.02E-11	1.09E-10	5.40E-01	2.01E-01	1.50E-02
F7	1.68E-03	4.31E-08	3.02E-11	6.35E-02	1.99E-02	6.53E-08	9.21E-05
F8	1.32E-04	8.19E-01	3.02E-11	8.77E-02	6.07E-11	3.02E-11	1.08E-02
F9	4.06E-02	4.36E-02	4.98E-11	2.92E-09	8.15E-11	3.02E-11	8.68E-03
F10	3.02E-11	1.55E-09	3.02E-11	1.91E-02	2.15E-10	5.30E-01	3.02E-11
F11	2.23E-09	7.38E-10	3.02E-11	3.02E-11	1.53E-05	3.02E-11	4.31E-08
F12	5.57E-10	6.12E-10	3.02E-11	1.46E-10	1.47E-07	3.34E-11	6.12E-10
F13	5.26E-04	4.64E-03	3.02E-11	3.37E-05	1.21E-10	4.62E-10	7.74E-06
F14	3.02E-11	3.02E-11	3.02E-11	3.02E-11	7.17E-01	1.01E-08	2.37E-10
F15	2.16E-03	1.78E-10	3.02E-11	5.07E-10	4.92E-01	1.89E-04	1.75E-05
F16	3.63E-01	5.27E-05	3.02E-11	4.46E-01	6.73E-01	1.34E-05	2.67E-09
F17	2.89E-03	4.64E-05	3.02E-11	9.82E-01	2.38E-07	3.83E-05	7.38E-10
F18	3.20E-09	2.37E-10	3.02E-11	8.99E-11	5.40E-01	3.69E-11	1.09E-10
F19	2.46E-01	2.39E-04	3.02E-11	4.71E-04	7.12E-09	1.02E-05	9.88E-03
F20	1.91E-02	4.08E-11	3.02E-11	3.50E-03	9.00E-01	8.53E-01	1.01E-08
F21	2.71E-01	2.23E-01	3.02E-11	9.92E-11	1.39E-06	6.72E-10	5.69E-01
F22	2.13E-05	4.50E-11	3.34E-11	2.92E-09	1.33E-01	1.31E-08	5.69E-01
F23	1.11E-06	3.02E-11	3.02E-11	8.24E-02	1.86E-01	8.35E-08	5.20E-01
F24	1.20E-08	7.22E-06	3.02E-11	3.02E-11	2.77E-05	3.02E-11	1.49E-04
F25	2.83E-08	1.60E-07	3.02E-11	3.47E-10	1.10E-08	1.77E-03	6.95E-01
F26	4.83E-01	4.08E-11	5.57E-10	1.10E-08	1.10E-08	1.26E-01	1.07E-09
F27	1.07E-09	2.38E-07	3.02E-11	3.02E-11	4.86E-03	3.02E-11	1.17E-09
F28	1.03E-06	3.69E-11	3.02E-11	5.49E-11	1.68E-03	8.99E-11	6.53E-07
F29	8.89E-10	3.02E-11	3.02E-11	3.02E-11	9.63E-02	3.02E-11	3.34E-11

**Table 7:** Statistical results of MEDBO compared with other improved DBO algorithms (Dim = 30).

		Dim = 30					
		**MSDBO**	**QOLDBO**	**SCDBO**	**GODBO**	**QHDBO**	**MEDBO**
F7	min	1.25E+05	5.31E+02	9.02E+02	7.22E+02	8.43E+02	7.00E+02
	mean	9.61E+05	7.44E+02	9.38E+02	7.44E+02	9.12E+02	**7.02E+02**
	std	6.78E+05	7.65E+06	7.66E+02	1.09E+03	3.30E+01	2.91E+03
	degree	6	3	5	2	4	1
F12	min	3.97E+03	4.88E+04	4.11E+03	4.66E+04	2.94E+04	9.29E+03
	mean	4.97E+04	6.93E+04	4.66E+04	7.04E+04	4.79E+04	**4.61E+04**
	std	6.46E+07	1.43E+14	3.33E+09	9.47E+09	3.31E+08	1.46E+09
	degree	4	5	2	6	3	1
F19	min	2.24E+03	2.37E+03	2.25E+03	6.41E+03	2.39E+03	2.30E+03
	mean	2.97E+03	4.01E+03	2.49E+03	7.05E+03	2.47E+03	**2.47E+03**
	std	4.45E+04	6.46E+12	3.76E+04	3.64E+06	2.21E+02	6.53E+04
	degree	4	5	3	6	2	1
F26	min	2.98E+03	3.12E+03	3.21E+03	2.80E+03	2.90E+03	2.93E+03
	mean	3.44E+03	3.19E+03	3.25E+03	3.31E+03	5.20E+03	**2.98E+03**
	std	7.61E+04	2.21E+02	8.21E+03	4.32E+03	3.54E+02	2.95E+03
	degree	5	2	3	4	6	1

**Table 8:** Statistical results of MEDBO compared with other improved DBO algorithms (Dim = 100).

		Dim = 100					
		**MSDBO**	**QOLDBO**	**SCDBO**	**GODBO**	**QHDBO**	**MEDBO**
F7	min	1.44E+06	1.61E+03	1.71E+03	1.91E+03	1.65E+03	1.70E+03
	mean	6.45E+06	2.66E+03	2.21E+03	2.64E+03	2.84E+03	**2.16E+03**
	std	9.56E+08	4.65E+05	8.46E+03	4.32E+03	1.68E+02	4.91E+04
	degree	6	4	2	3	5	1
F12	min	7.45E+04	4.89E+05	8.29E+04	3.21E+07	8.44E+07	8.02E+06
	mean	8.45E+08	7.45E+08	**6.64E+05**	6.45E+08	5.63E+08	9.63E+07
	std	6.64E+13	1.38E+09	1.56E+05	1.45E+14	1.73E+09	1.15E+16
	degree	6	5	1	4	3	2
F19	min	4.76E+03	5.13E+03	5.21E+03	6.43E+03	6.11E+03	5.01E+03
	mean	5.50E+03	5.79E+03	6.37E+03	6.11E+03	6.53E+03	**5.41E+03**
	std	1.56E+05	6.45E+07	3.58E+04	7.59E+04	5.79E+02	5.56E+05
	degree	2	3	5	4	6	1
F26	min	3.92E+03	3.79E+03	3.56E+03	3.13E+03	9.45E+03	3.41E+03
	mean	5.46E+03	4.46E+03	3.71E+03	6.12E+03	1.99E+04	**4.30E+03**
	std	9.56E+06	1.97E+06	2.38E+05	4.48E+03	1.47E+03	6.23E+04
	degree	4	3	2	5	6	1

**Table 9:** Statistical measurement analysis of the tension (compression) spring design.

	COA	HHO	BOA	OMA	WOA	SAO	DBO	MEDBO
mean	0.013120	0.013597	0.013193	0.012765	0.012666	0.013790	0.013796	0.012665
std	3.1E-07	1.05E-06	1.23E-08	5.84E-09	7.91E-13	2.71E-06	2.55E-06	4.53E-05
min	0.012667	0.012666	0.013093	0.012674	0.012665	0.012703	0.012686	0.012665
max	0.014757	0.017206	0.013993	0.012967	0.012669	0.017773	0.017781	0.012669
ranking	4	6	5	3	2	7	8	1

**Table 10:** Statistical measurement analysis of the triangle truss design.

	COA	HHO	BOA	OMA	WOA	SAO	DBO	MEDBO
mean	263.8990	264.0097	274.8039	263.8960	263.8959	263.8970	263.9041	263.8959
std	9.41E-06	1.14E-02	3.03E+01	3.85E-08	6.69E-28	2.36E-06	3.53E-04	1.43E-08
min	263.8960	263.8989	265.2901	263.8958	263.8958	263.8958	263.8958	263.8958
max	263.9081	264.2654	282.8427	263.8968	263.8961	263.9023	263.9964	263.8981
ranking	5	7	8	3	2	4	6	1

**Table 11:** Statistical measurement analysis of the triangle truss design.

	COA	HHO	BOA	OMA	WOA	SAO	DBO	MEDBO
mean	6518.473	6822.387	557,202.6	5964.435	5889.333	6454.828	6661.39	5889.249
std	2.70E+05	1.99E+05	2.74E+11	3.32E+03	6.24E-11	2.75E+05	3.48E+06	2.76E-05
min	5892.604	6089.84	76052.22	5887.563	5885.333	5889.434	5885.333	5885.333
max	7323.958	7753.804	2532499	6076.547	5899.333	7319.001	16,043.15	7319.001
ranking	5	7	8	3	2	4	6	1

## Data Availability

The original contributions presented in the study are included in the article, further inquiries can be directed to the corresponding authors.
